# Amhy/Amhr2y-mediated sex determination in two distantly related teleosts relies on the conserved Alk3-Smad5 axis

**DOI:** 10.1093/molbev/msag038

**Published:** 2026-02-09

**Authors:** Li Zhou, Liang Zhang, Zeting Qu, Hongqin Jian, Shuqing Zheng, Minghui Li, Deshou Wang, Xingyong Liu

**Affiliations:** Integrative Science Center of Germplasm Creation in Western China (Chongqing) Science City, Key Laboratory of Freshwater Fish Reproduction and Development (Ministry of Education), Key Laboratory of Chongqing Municipality for Aquatic Economic Animal Resources Conservation and Germplasm Creation, Chongqing Technology Innovation Center of Breeding, School of Life Sciences, Southwest University, Chongqing 400715, China; Integrative Science Center of Germplasm Creation in Western China (Chongqing) Science City, Key Laboratory of Freshwater Fish Reproduction and Development (Ministry of Education), Key Laboratory of Chongqing Municipality for Aquatic Economic Animal Resources Conservation and Germplasm Creation, Chongqing Technology Innovation Center of Breeding, School of Life Sciences, Southwest University, Chongqing 400715, China; Integrative Science Center of Germplasm Creation in Western China (Chongqing) Science City, Key Laboratory of Freshwater Fish Reproduction and Development (Ministry of Education), Key Laboratory of Chongqing Municipality for Aquatic Economic Animal Resources Conservation and Germplasm Creation, Chongqing Technology Innovation Center of Breeding, School of Life Sciences, Southwest University, Chongqing 400715, China; Integrative Science Center of Germplasm Creation in Western China (Chongqing) Science City, Key Laboratory of Freshwater Fish Reproduction and Development (Ministry of Education), Key Laboratory of Chongqing Municipality for Aquatic Economic Animal Resources Conservation and Germplasm Creation, Chongqing Technology Innovation Center of Breeding, School of Life Sciences, Southwest University, Chongqing 400715, China; Integrative Science Center of Germplasm Creation in Western China (Chongqing) Science City, Key Laboratory of Freshwater Fish Reproduction and Development (Ministry of Education), Key Laboratory of Chongqing Municipality for Aquatic Economic Animal Resources Conservation and Germplasm Creation, Chongqing Technology Innovation Center of Breeding, School of Life Sciences, Southwest University, Chongqing 400715, China; Integrative Science Center of Germplasm Creation in Western China (Chongqing) Science City, Key Laboratory of Freshwater Fish Reproduction and Development (Ministry of Education), Key Laboratory of Chongqing Municipality for Aquatic Economic Animal Resources Conservation and Germplasm Creation, Chongqing Technology Innovation Center of Breeding, School of Life Sciences, Southwest University, Chongqing 400715, China; Integrative Science Center of Germplasm Creation in Western China (Chongqing) Science City, Key Laboratory of Freshwater Fish Reproduction and Development (Ministry of Education), Key Laboratory of Chongqing Municipality for Aquatic Economic Animal Resources Conservation and Germplasm Creation, Chongqing Technology Innovation Center of Breeding, School of Life Sciences, Southwest University, Chongqing 400715, China; Integrative Science Center of Germplasm Creation in Western China (Chongqing) Science City, Key Laboratory of Freshwater Fish Reproduction and Development (Ministry of Education), Key Laboratory of Chongqing Municipality for Aquatic Economic Animal Resources Conservation and Germplasm Creation, Chongqing Technology Innovation Center of Breeding, School of Life Sciences, Southwest University, Chongqing 400715, China

**Keywords:** teleosts, sex-determining gene, Amhy/Amhr2y, type I receptor, R-Smad

## Abstract

In teleosts, homologs of Anti-Müllerian Hormone (Amhy) and its type II receptor (Amhr2/Amhr2y) have been independently recruited as master sex-determination genes in about 50% of known cases. However, it remains unknown whether a conserved transducer pair exists, as the requisite type I receptors and R-Smad effectors remain unidentified amidst their diversity and potential redundancy. In this study, we employed an in vitro reporter assay to screen five type I receptors (Alk2a, Alk2b, Alk3, Alk6a, Alk6b) and three R-Smads (Smad1, Smad5, Smad8), discovering that only Alk3, Alk6a, or Alk6b, in combination with Smad5, significantly activated Amhy/Amhr2 signaling. In Nile tilapia, levels of phosphorylated Smad5 (p-Smad5) were notably elevated in XY gonads compared with XX gonads during the critical sex-determination window (8 to 15 dpf), while total Alk3 and Smad5 expression did not exhibit sexual dimorphism. The inhibition of type I receptors in XY fish resulted in feminization or complete sex reversal. Similarly, CRISPR/Cas9 mutagenesis of *alk3* or *smad5* led to male-to-female sex reversal in F0 mosaic mutants. Importantly, homozygous mutations in *alk3* or *smad5* resulted in embryonic lethality at the gastrula stage, whereas mutations in other type I receptors or R-Smads were viable and demonstrated normal sexual development. The conservation of this pathway was further substantiated in Southern catfish, where mutations in *alk3a* or *smad5* also induced sex reversal in XY individuals. Collectively, our findings establish Alk3 and Smad5 as essential and specific transducers of the Amhy/Amhr2-mediated sex-determination pathway, revealing a potentially conserved signaling axis across teleosts.

## Introduction

Sex determination mechanisms in vertebrates, particularly in teleosts, exhibit remarkable diversity (Mank and Avise 2009; Cutting et al. 2013; Rovatsos et al. 2019; Nagahama et al. 2021; Pan et al. 2021; Curzon et al. 2023; Kitano et al. 2024). This variation is primarily driven by the repeated and independent evolution of master sex-determining genes (SDGs) across different lineages (van Doorn 2014; Herpin and Schartl 2015; Pennell et al. 2018; Nagahama et al. 2021; Li et al. 2022; Zhu et al. 2025). To date, SDGs have been identified in over 150 teleost species, representing a wide array of gene families. Notably, approximately 60% of these species utilize ligands or receptors from the TGF-β superfamily as their SDGs (Yu et al. 2024; Zhou et al. In press), highlighting a dominant evolutionary pathway in teleost sex determination. Within this superfamily, components of the Amh pathway emerge as significant contributors. Ligand-encoding genes such as *amhy* and *amhby*, along with receptor genes including *amhr2* and *amhr2y*, have been recurrently co-opted as SDGs. Remarkably, *amh* and *amhr2* homologs alone account for about 50% of all known SDG-bearing teleost species (Kitano et al. 2024; Wang et al. 2024; Zhou et al. In press), indicating a clear evolutionary preference. This trend sharply contrasts with the limited role of Amh signaling in tetrapod sex determination, which has only been tentatively reported in a few monotreme and reptile species (Cortez et al. 2014; Rovatsos et al. 2019; Shearwin-Whyatt et al. 2025). With ongoing genomic discoveries, the identification of additional teleost species employing *amh*/*amhr2* homologs as SDGs is highly anticipated.

Anti-Müllerian hormone (Amh), also known as Müllerian inhibiting substance, is a ligand of the TGF-β superfamily that signals through its specific type II receptor, Amhr2—a partnership conserved across mammals and teleosts (Visser 2003; Shiraishi et al. 2008; Rocha et al. 2016; Mullen et al. 2019; Liu et al. 2020; Yamaguchi and Kitano 2023). In tetrapods, its canonical role is to induce the regression of the Müllerian duct in male embryos, thereby promoting the development of the male reproductive tract (Adolfi et al. 2019; Moses and Behringer 2019; Brunello and Rey 2022; Nelson et al. 2023). The canonical Amh signaling pathway is initiated when the Amh homodimer binds to Amhr2, which subsequently recruits and activates the type I receptor. This receptor complex then phosphorylates intracellular R-Smads. The phosphorylated R-Smads form a complex with Co-Smad, translocate to the nucleus, and regulate the transcription of target genes (Zhao et al. 2018; Hachana and Larrivée 2022). The specificity of the Amh-Amhr2 interaction is well-established, with research consistently identifying Amhr2 as the dedicated type II receptor for Amh signaling (Hart et al. 2021; Cate 2022). This finding has been robustly confirmed in Nile tilapia, where Amhr2 has been proven to be the exclusive receptor for Amhy-mediated sex determination (Liu et al. 2022).

In contrast to the specific ligand-receptor pairing observed with type II receptors, Amh signaling in tetrapods involves multiple candidate type I receptors, including Alk2 (Acvr1), Alk3 (Bmpr1a), and Alk6 (Bmpr1b) (Howard et al. 2022). The functional roles of these receptors are highly context-dependent, varying by cell type and biological process (Kloen et al. 2003; ten Dijke et al. 2003; Sahni et al. 2010; Hinck 2012; Lin et al. 2016; Mang et al. 2020). In Müllerian duct regression, Alk3 serves as the primary type I receptor. Conditional knockout of Alk3 in mice resulted in Müllerian duct retention in approximately 55% of males, while dual knockout of Alk2 and Alk3 led to complete retention in all individuals (Orvis et al. 2008). This observation highlights functional redundancy, with Alk2 compensating for the loss of Alk3. Spatiotemporal expression patterns further support their coordinated action: Alk2 is expressed earlier in the celomic epithelium, whereas Alk3 appears later and is restricted to the mesenchyme, suggesting a sequential involvement in Müllerian duct regression (Zhan et al. 2006). Consistent with this, both Tlx-2 reporter assays and Müllerian duct organ culture have confirmed the essential role of Alk2 in Amh-induced signaling (Visser et al. 2001). Similar compensatory interactions occur in other cell types. In immature Sertoli cells of mice, Alk3 mediates Amh signaling through Smad1 phosphorylation; however, Alk2 is capable of transducing the signal in the absence of Alk3 (Belville et al. 2005). In human theca cells, Amh suppresses Cyp17a1 expression and androgen synthesis—a process potentially mediated by Alk2 and Alk5 (Chen et al. 2023). Beyond Müllerian duct regression, Alk6 functions as the principal type I receptor in regulating ovulation rate and the estrous cycle, as demonstrated in mice, sheep, pig and human (Souza et al. 2001; Yi et al. 2001; Regan et al. 2016; Liu et al. 2025). Together, these findings illustrate that the mammalian Amh signaling pathway engages distinct type I receptors in a cell- and process-specific manner, often accompanied by functional complementation among these receptors.

Following the teleost-specific third round of whole-genome duplication, the repertoire of type I receptor genes has expanded (Zheng et al. 2018; Liu et al. 2023). For example, the Nile tilapia genome contains five such genes (*alk2a*, *alk2b*, *alk3*, *alk6a*, *alk6b*), whereas the Southern catfish (*Silurus meridionalis*) genome includes *alk2a*, *alk2b*, *alk3a*, *alk3b*, and *alk6* (Zheng et al. 2022). However, the functional roles of these receptors in the Amh signaling pathway remain poorly understood. To date, Alk6b (Bmpr1bb) is the only type I receptor with a clearly defined function: it has been identified as a candidate SDG in Atlantic herring (*Clupea harengus*) and has also been shown to mediate Amh-regulated germ cell proliferation in zebrafish (Neumann et al. 2011; Rafati et al. 2020). Consequently, despite the prevalence of Amh and Amhr2 homologs as SDGs in teleosts, the specific type I receptor(s) required for this crucial signaling pathway await identification.

At the intracellular signaling level, the Amh pathway relies on R-Smad proteins (Smad1, Smad5, and Smad8) to transduce its signal (Josso and Clemente 2003; Visser 2003; Visser and Themmen 2005; Cate 2022; Howard et al. 2022; Josso and Picard 2022). The receptor complex formed by the Amh type II and type I receptors on the cell membrane recruits and phosphorylates R-Smad proteins. Studies in mammals have revealed functional specialization and compensatory mechanisms among different R-Smads in Amh signaling (Kaivo-oja et al. 2006; Pangas 2012; Itman and Loveland 2013; Li 2015). Research in mice has shown that in the canonical Amh pathway responsible for Müllerian duct regression, Smad5 serves as the primary mediator, while Smad1 and Smad8 exhibit functional compensation (McReynolds et al. 2007; Conidi et al. 2011; Costamagna et al. 2016; Rodriguez et al. 2016; Monsivais et al. 2021; Tang et al. 2022). Further studies in geese indicate that Smad8 acts as the principal R-Smad in the Amh pathway regulating follicle selection and recruitment (Yu et al. 2019), highlighting the biologically process-specific roles of R-Smads. In teleosts, although one copy of each of the three R-Smad genes is retained, their functions in Amh signaling remain poorly understood. Current in vitro evidence from Nile tilapia suggests that Amhy/Amhr2 can inhibit the promoter activity of *cyp19a1a* through the phosphorylation of Smad1/5/8 (Liu et al. 2022), implying that this pathway may influence sex determination by regulating estrogen levels via the Smad-dependent pathway. However, these findings still require validation through in vivo functional experiments, and it remains unclear which R-Smad plays the dominant role.

Therefore, despite the identification of *amh*/*amhr2* homologs as SDGs in multiple teleosts species, the specific type I receptors and R-Smad molecules involved in their downstream signaling remain a critical unresolved question. This study aims to elucidate the key type I receptor and R-Smad factor in the Amhy/Amhr2y-mediated sex-determination pathway. Using Nile tilapia and Southern catfish as models, we combined in vitro luciferase reporter assays with in vivo functional validation through inhibitor treatments and CRISPR/Cas9 gene editing. Our findings provide new insights into the molecular basis and evolutionary conservation of sex determination mechanisms in teleosts.

## Results

### Specific type I receptor and R-Smad combinations mediate Amhy/Amhr2 signaling

**Figure 1:**
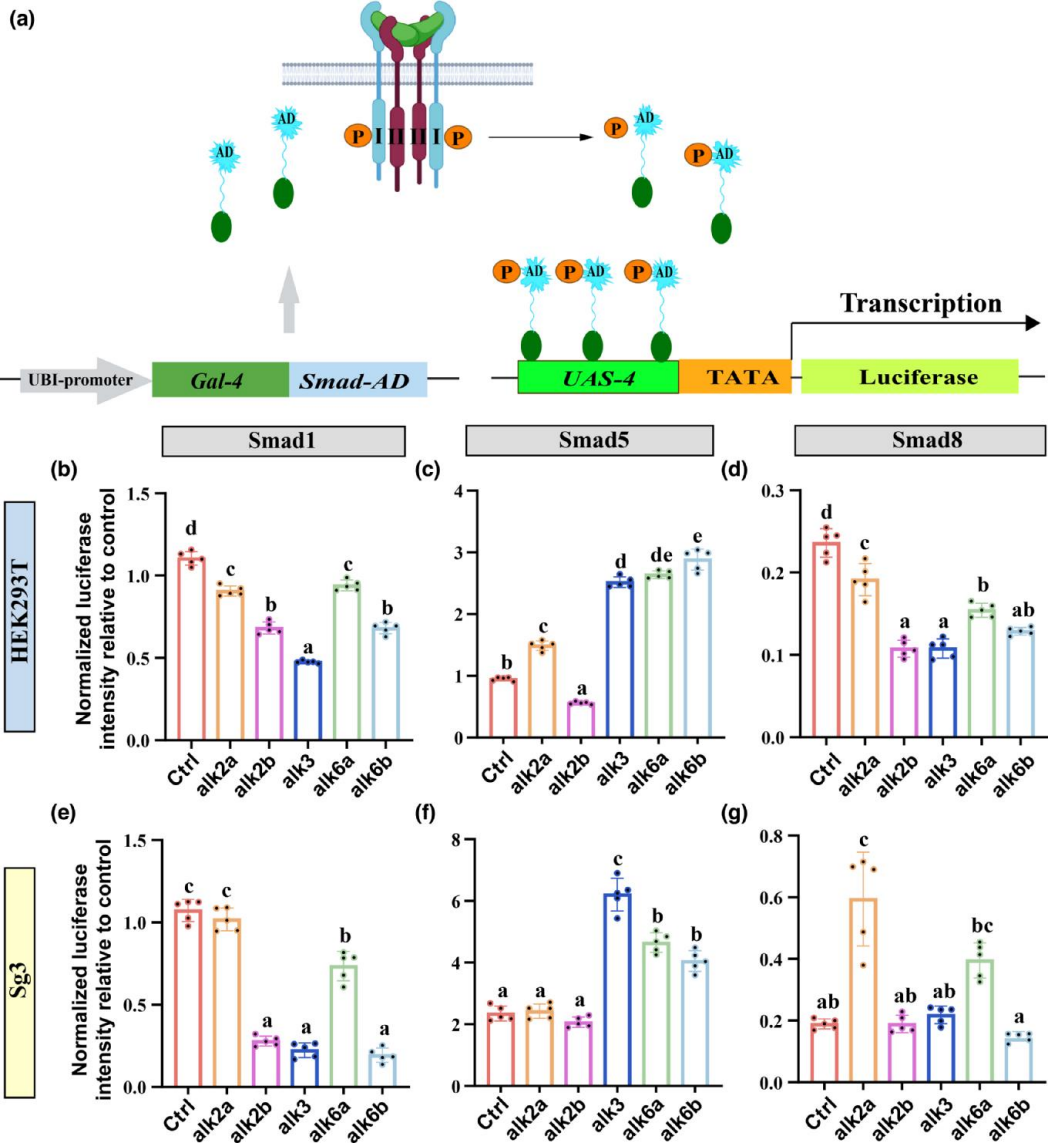
Assessment of Amh Signaling through a Smad Phosphorylation Reporter Assay in HEK293T and SG3 cells. a) Diagram of the UAS-Gal4-Smad-AD reporter assay for quantifying Amh-induced Smad1/5/8 phosphorylation (see Materials and methods). In this system, ligand-receptor binding triggers phosphorylation and nuclear translocation of the Gal4-Smad-AD fusion protein. This enables binding to the UAS promoter via the Gal4 domain, activating luciferase expression. Luciferase activity thereby correlates directly with Smad phosphorylation levels, reporting specific upstream signaling. “AD” refers to the transactivation domain of individual R-Smads. (b–g) Normalized luciferase activity in HEK293T b–d) and SG3 e–g) cells transfected with Amh, Amhr2, an R-Smad, and different type I receptors. Values are expressed relative to the control (transfected with Amh, Amhr2, and R-Smad).

To delineate the Amhy signaling cascade in Nile tilapia, we investigated the roles of type I receptors and R-Smad proteins using a luciferase reporter assay. We co-transfected medaka SG3 and HEK293T cells with Nile tilapia Amhy, Amhr2, various type I receptors, and transcriptional activation plasmids for Smad1, Smad5, or Smad8. Signaling activity, indicative of R-Smad phosphorylation, was quantified by measuring luciferase activity (Fig. 1a). In assays evaluating Smad1 activation, none of the type I receptors enhanced phosphorylation in either cell line (Fig. 1b, e). In contrast, Alk3, Alk6a, and Alk6b significantly increased Smad5 phosphorylation in both SG3 and HEK293T cells (Fig. 1c, f). For Smad8, no type I receptor enhanced its phosphorylation in HEK293T cells; however, Alk2a did enhance it in SG3 cells (Fig. 1d, g).

### Inhibition of the type I receptor induces male-to-female sex reversal in Nile tilapia

**Figure 2:**
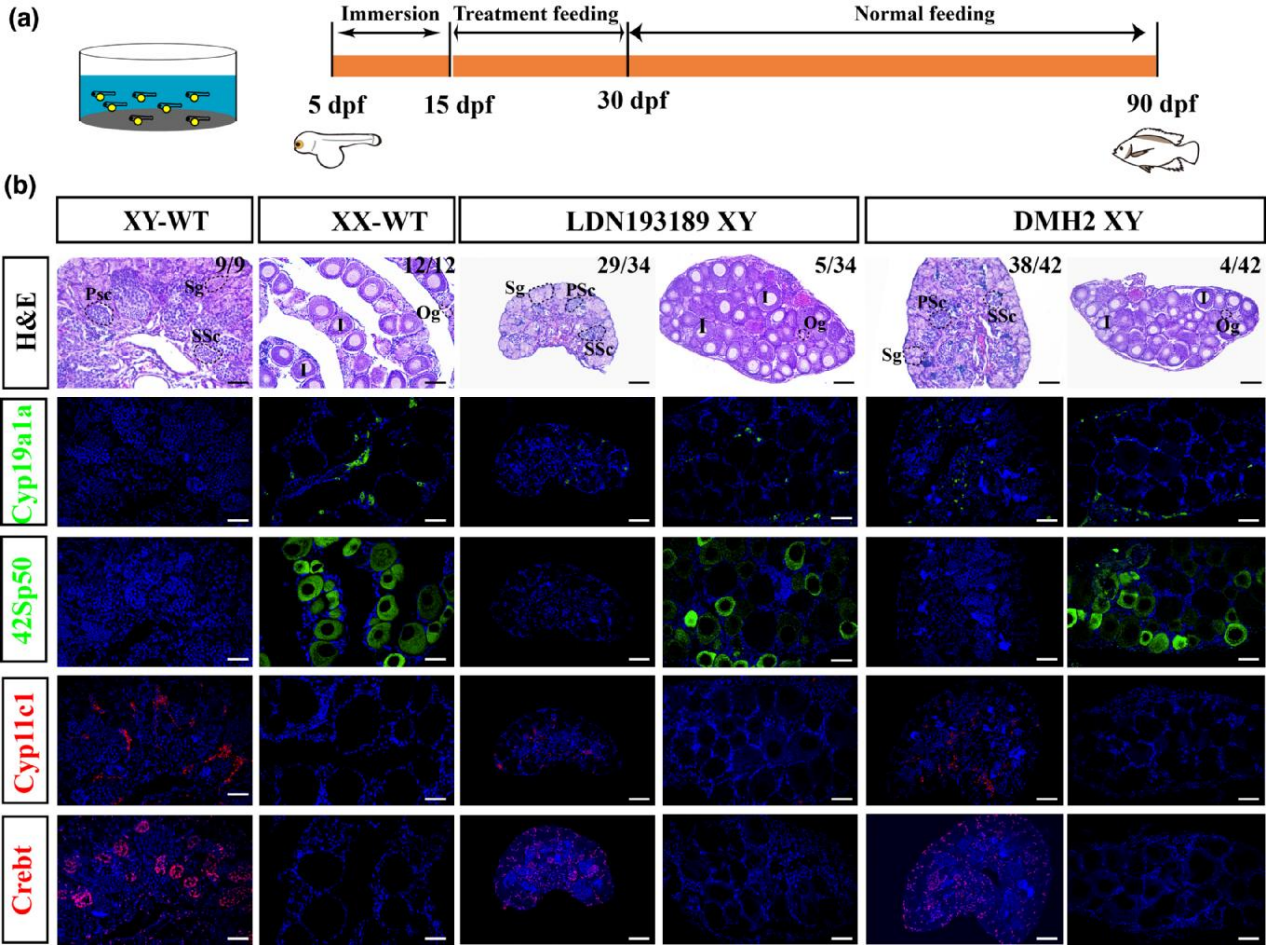
Inhibition of Bmp type I receptors disrupts male sex determination in Nile tilapia. a) Experimental timeline of inhibitor treatment. From 5 to 15 days post-fertilization (dpf), fish were exposed to DMH2 (200 nM) or LDN193189 (250 nM) via water immersion. From 15 to 30 dpf, the same inhibitors were administered via a supplemented diet. All fish were subsequently raised on a normal commercial diet until sampling at 90 dpf. b) Histological analysis of gonads at 90 dpf. Sections from XY-WT, XX-WT, LDN193189-treated XY, and DMH2-treated XY fish are shown. Sg, spermatogonia; PSc, primary spermatocytes; SSc, secondary spermatocytes; Og, oogonia; I, phase I follicle. Immunofluorescence (IF) analysis of gonadal sex markers. Expression of testis-dominant genes (Cyp11c1, Crebt) and ovary-dominant genes (Cyp19a1a, 42Sp50) was examined in the corresponding groups. Scale bar = 25 μm.

To investigate the role of type I receptors in Smad-dependent sex determination in Nile tilapia, XY fish were treated with two type I receptor inhibitors: LDN193189, an inhibitor of the Bmp type I receptors, and DMH2, which primarily targets Alk3 signaling. The XY fish (5 days post-fertilization, dpf) underwent a 10-day immersion treatment, followed by 15 days of feeding with inhibitor-containing feed, and were then raised on normal feed until sampling at 90 dpf (Fig. 2a). Histological analysis revealed that all control XY fish developed as males with typical testes. In contrast, a subset of LDN193189-treated XY fish (5 of 34) underwent sex reversal, developing as females with ovaries, while the majority (29 of 34) developed as males but exhibited delayed gonadal development. Similarly, DMH2 treatment also induced sex reversal in a portion of XY fish (4 of 42). Fluorescence immunohistochemistry was employed to assess the expression of ovary-dominant genes (Cyp19a1a and 42Sp50) and testis-dominant genes (Cyp11c1 and Crebt). In inhibitor-treated XY fish, Cyp19a1a expression was upregulated in the ovaries of sex-reversed individuals. Even in those that developed as males, weak expression of Cyp19a1a was detected in the testes (Fig. 2b). Positive signals for the oocyte marker 42Sp50 were observed in the ovaries of sex-reversed XY fish, similar to wild-type XX (WT-XX) females (Fig. 2b). In contrast, the gonads of untreated control XY fish expressed Cyp11c1 and Crebt, whereas no positive signals for these markers were detected in sex-reversed individuals (Fig. 2b). These results demonstrate that inhibition of type I receptors disrupts Amh signaling, leading to feminization or complete sex reversal in XY Nile tilapia.

### P-Smad5 shows a sexually dimorphic expression pattern during the critical period for Nile tilapia sex determination

**Figure 3:**
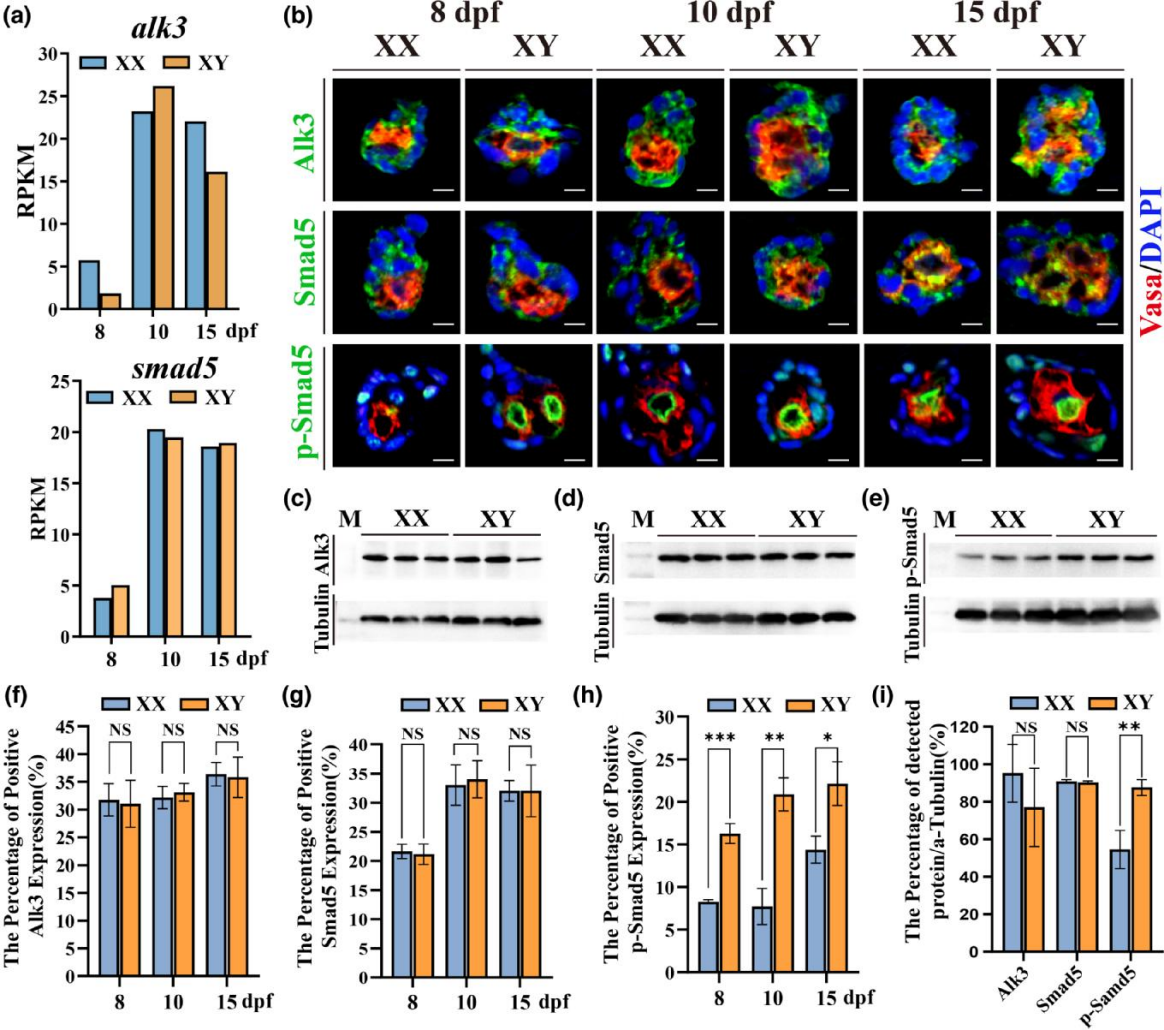
Expression profiles of Alk3, Smad5, and p-Smad5 during sex determination in gonads of Nile tilapia. a) Transcriptomic data (Tao et al. 2013) showing the expression of *alk3* and *smad5* in gonads during sex determination. No sexually dimorphic expression was detected between XY and XX fish at 8, 10, and 15 dpf. b, f–h) Immunofluorescence (IF) analysis of Alk3, Smad5, and p-Smad5 expression. Quantitative assessment of fluorescence signals revealed no significant differences in Alk3 and Smad5 levels between XX and XY tilapia gonads at 8, 10, and 15 dpf. In contrast, p-Smad5 signals were significantly stronger in XY fish compared with XX fish. Green indicates Alk3, Smad5, or p-Smad5; red denotes the germ cell marker Vasa. c–e, i) Western blot analysis confirmed that total protein levels of Alk3 and Smad5 did not differ significantly between sexes, whereas the total p-Smad5 protein level was significantly higher in XY fish than in XX fish at 10 dpf.

Our data from luciferase reporter and inhibitor assays, combined with gene mutation analysis, lead us to hypothesize that Alk3 and Smad5 are essential transducers of the Amhy sex-determination signal in Nile tilapia. We initially examined their spatiotemporal expression profiles. Analysis of the transcriptomic dataset published by our group (Tao et al. 2013) and validated by real-time PCR revealed that none of the *alks* and *smads* exhibited sexually dimorphic mRNA expression between female and male gonads during the critical period for sex determination (8 to 15 dpf) (Fig. 3a, Figure S1). Subsequently, we generated polyclonal antibodies against tilapia Alk3 and Smad5, and employed a commercial anti-p-Smad5 antibody for double fluorescence immunohistochemistry on gonads at 8, 10, and 15 dpf. Alk3 and Smad5 were detected in both somatic and germ cells at all stages, primarily localized in the cytoplasm and partially overlapping with the germ cell marker Vasa, without clear sexual dimorphism. In contrast, p-Smad5 was present in the nuclei of somatic and germ cells and did not colocalize with Vasa. Notably, p-Smad5 was already abundant in the germ cell nuclei of XY males by 8 dpf, while it was faint in XX females at this stage, increasing by 10 dpf (Fig. 3b, f–h). Western blot analysis of gonadal protein extracts at 10 dpf further demonstrated that total Alk3 and Smad5 protein levels did not differ between sexes (Fig. 3c, d, i). However, p-Smad5 levels were significantly higher in XY gonads compared with XX gonads (Fig. 3e, i), supporting a sexually dimorphic activation pattern during the critical window of sex determination.

### Disruption of Nile tilapia *alk3* or s*mad5* leads to male-to-female sex reversal

**Figure 4:**
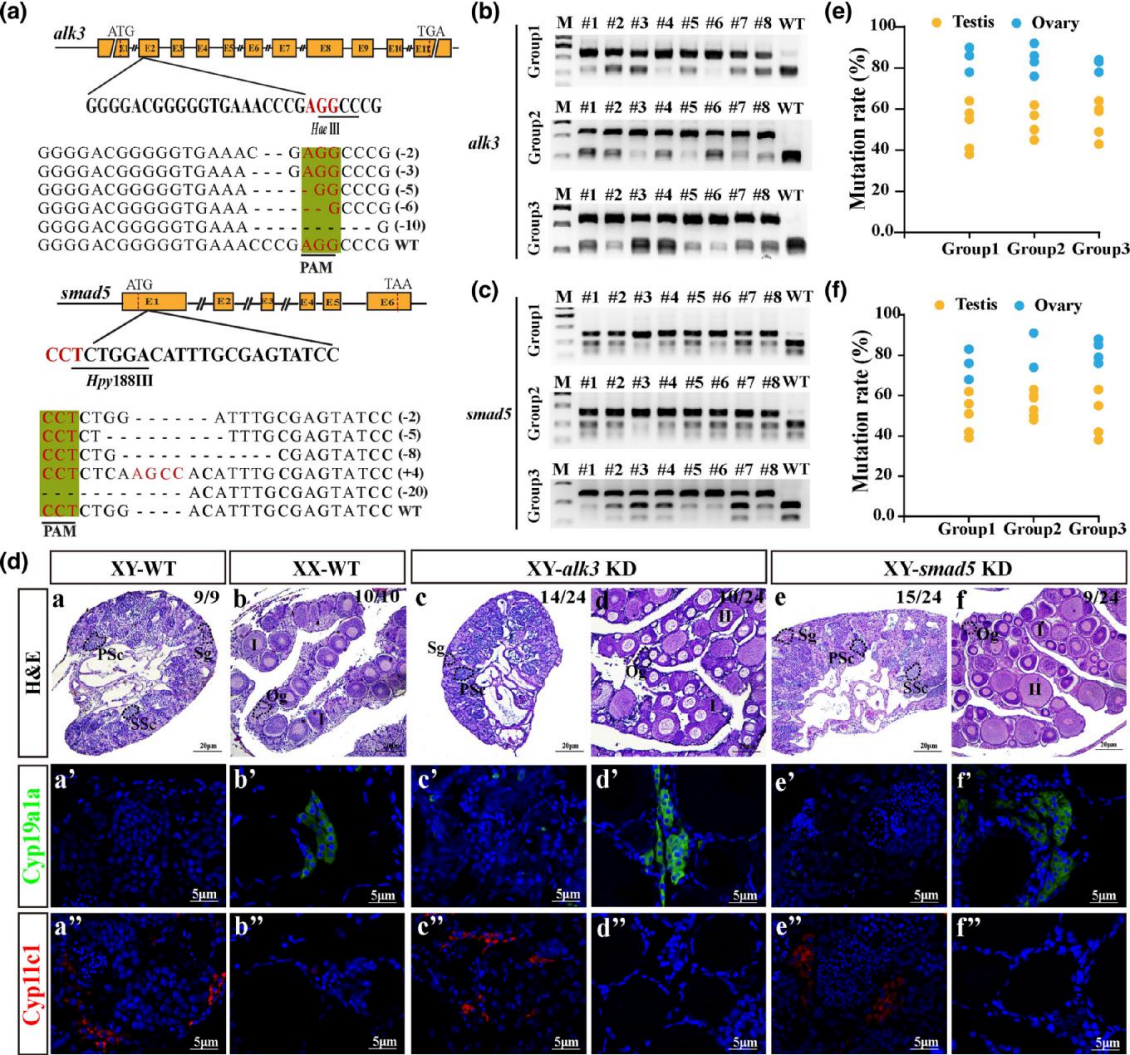
CRISPR/Cas9-mediated knockdown of *alk3* or *smad5* disrupts male sex determination in XY tilapia. a) Schematic representation of the guide RNA (gRNA) target sites designed for the knockdown (KD) of the *alk3* and *smad5* genes, along with the representative mutation types identified in the F0 generation. b, c) Identification of positive F0 XY fish. High-efficiency mutants for *alk3* b) and *smad5* c) were identified using restriction enzyme digestion and agarose gel electrophoresis, with mutation screening conducted across more than three independent biological replicates. d) Histological and immunofluorescence (IF) analysis of gonads from *alk3* and *smad5* mutant XY fish at 90 dpf. IF staining was performed to assess the expression of testis-dominant genes (Cyp11c1) and ovary-dominant genes (Cyp19a1a). Sg, spermatogonia; PSc, primary spermatocytes; SSc, secondary spermatocytes; Og, oogonia; I to II, phase I to II follicle. e, f) Quantification of mutation efficiency and its correlation with the sex reversal phenotype. The incidence of sex reversal was observed in *alk3* and *smad5* mutant fish, with mutation efficiencies exceeding 70% and 63.5%, respectively.

To further validate our hypothesis, we utilized CRISPR/Cas9 technology to generate mutations in the *alk3* and *smad5* genes in XY Nile tilapia. Targeting sites were strategically designed within the second exon of *alk3* and the first exon of *smad5* (Fig. 4a). Sequencing of F0 mutants confirmed the presence of various frameshift and nonframeshift mutations at both loci. Multiple independent gene editing experiments were conducted for each gene. Positive mutants were identified at 90 dpf using restriction enzyme digestion (*Hae*III for *alk3* and *Hpy*188III for *smad5*; Fig. 4b, c). Gonadal histological analysis was performed on three randomly selected groups of mutants for each gene. In the *alk3* XY mutants, a subset of individuals with high mutation rates (3/8, 4/8, 3/8 per group) underwent sex reversal, developing ovaries, whereas nonmutated XY fish from the same groups developed testes. Similarly, a proportion of *smad5* XY mutants (3/8, 2/8, 4/8) also exhibited sex reversal and formed ovaries, while their nonmutant XY counterparts developed testes (Fig. 4d). Mutation rate analysis indicated gonadal editing efficiencies exceeding 70% in sex-reversed *alk3* mutants and over 63.5% in sex-reversed *smad5* mutants (Fig. 4e, f). Immunofluorescence analysis revealed strong expression of Cyp19a1a and the absence of Cyp11c1 in the gonads of sex-reversed mutants. In contrast, nonsex-reversed mutants exhibited low or undetectable levels of Cyp19a1a but high expression of Cyp11c1 (Fig. 4d). These results confirm that the disruption of *alk3* or *smad5* induces male-to-female sex reversal in XY Nile tilapia. Concurrently, we assessed p-Smad5 levels in *alk3* mutants at 30 dpf, the p-Smad5 levels were found to be significantly reduced compared with those in XY-WT fish, indicating that Alk3 is the key type I receptor mediating Smad5 phosphorylation within the Amhy/Amhr2 signaling pathway in tilapia sex determination (Figure S2).

### Homozygous mutants of *alk3* and *smad5* were embryonic lethality in Nile tilapia

**Figure 5:**
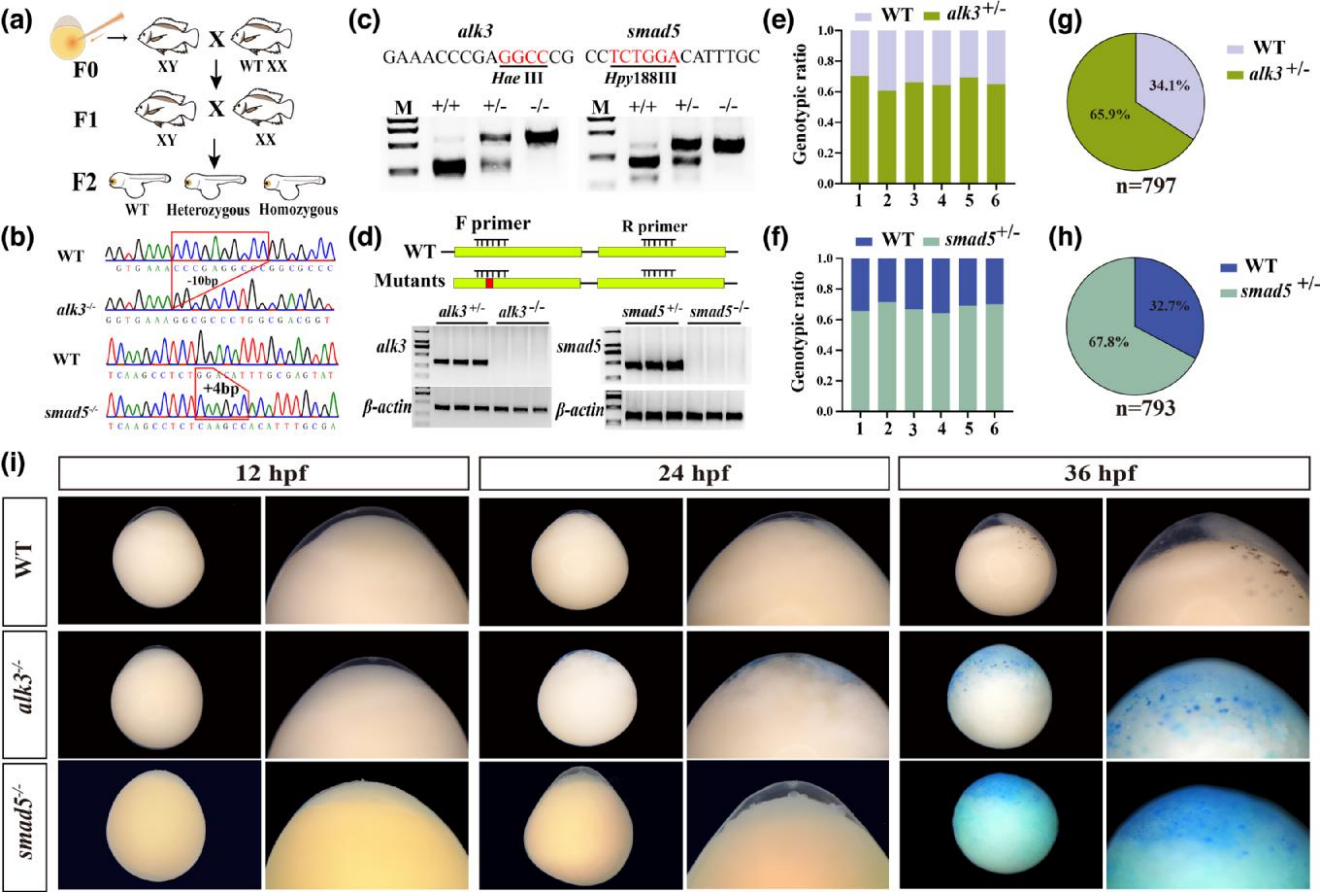
Homozygous mutation of *alk3* or *smad5* on embryonic development in Nile tilapia. a) Workflow for generating *alk3* and *smad5* homozygous mutant lines. b) Selection and verification of founder (F0) mutants. Shown are the sequences of the selected F0 mutants carrying a 10-bp deletion (*alk3*) or a 4-bp insertion (*smad5*) used to establish the stable lines. c, d) Genotypic identification of homozygous mutants. Restriction enzyme digestion assay of F2 embryos c). RT-PCR validation using a forward primer spanning the mutation-site (red box) d). No product was amplified from homozygous mutant cDNA, while a clear band was detected in WT-XY testes; β-actin served as the loading control. e–h) Genotypic distribution analysis. Statistical results of the genotypic ratios in the F2 generation from different *alk3* e, g) and *smad5* f, h) sibling crosses. i) Embryonic development of homozygous mutants. Both *alk3* and *smad5* homozygous mutants exhibited developmental abnormalities from 24 h post-fertilization (hpf) and arrested at the gastrula stage, resulting in embryonic lethality.

To verify that mutations in *alk3* or *smad5* induce male-to-female sex reversal in XY mosaic fish, we generated homozygous mutants for both genes. Given that males mature earlier than females, we crossed nonsex-reversed F0 XY mutants with wild-type XX females to produce the F1 generation. Sexually mature F1 individuals carrying frameshift mutations were subsequently intercrossed to obtain F2 homozygous mutants (Fig. 5a). Sequencing of F2 embryos confirmed a 10-bp deletion in *alk3* and a 4-bp insertion in *smad5* homozygous mutants (Fig. 5b). Restriction enzyme digestion and RT-PCR further validated the genotypes: wild-type DNA was completely digested, heterozygotes exhibited partial digestion, and homozygous mutant DNA remained undigested (Fig. 5c). Similarly, RT-PCR employing a mutation-site-specific primer successfully amplified products from heterozygous but not from homozygous embryos (Fig. 5d). Despite confirming the presence of homozygous mutants in F2 embryos, multiple screenings of 2-month-old F2 fish from various F1 crosses failed to identify any surviving *alk3* or *smad5* homozygotes. Genotyping analysis revealed that the ratios of wild-type to heterozygous mutants in F2 progeny were approximately 1:2 across several batches (Fig. 5e, f). Overall, heterozygotes comprised 65.9% (*alk3*, *n* = 797) and 67.8% (*smad5*, *n* = 793) of the F2 populations, with wild-type individuals making up the remainder (Fig. 5g, h). To ascertain the timing of lethality, we monitored embryonic development and discovered that homozygous mutants for both genes exhibited gastrulation defects, failed to complete epiboly, and succumbed by the gastrula stage (Fig. 5i). These results indicate that homozygous loss of *alk3* or *smad5* is embryonically lethal in Nile tilapia.

### Mutations in other type I receptors and Smads result in no sex reversal in Nile tilapia

**Figure 6:**
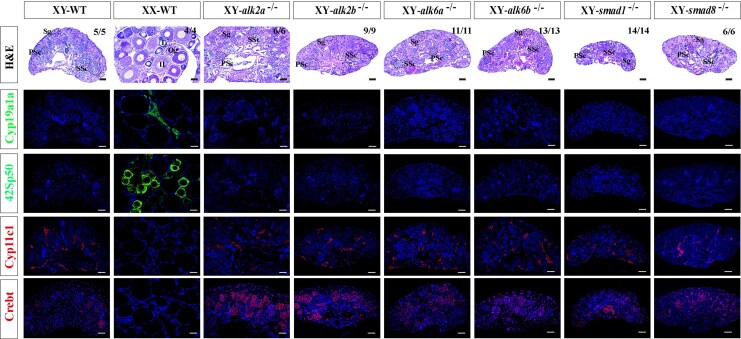
Gonadal phenotypes of XY mutants for other type I receptors and R-Smads in the Amh signaling pathway. Histological and immunofluorescence (IF) analysis of gonads from the indicated homozygous mutant lines at 90 dpf. All mutant XY fish developed normal testes without signs of sex reversal. IF analysis confirmed the presence of Cyp11c1 and Crebt, and the absence of Cyp19a1a and 42Sp50 in all mutants, indicating that male sex determination and differentiation proceeded normally. Sg, spermatogonia; PSc, primary spermatocytes; SSc, secondary spermatocytes; Og, oogonia; I to II, phase I to II follicle. Scale bar = 25 μm.

To investigate whether other type I receptors (Alk2a, Alk2b, Alk6a, Alk6b) and Smad factors (Smad1, Smad8) are involved in Amhy/Amhr2 signal transduction, we generated F0 mutants for each gene using CRISPR/Cas9 technology. Sanger sequencing, restriction enzyme digestion, and RT-PCR confirmed the successful mutation of all six genes; however, no sex reversal was observed in XY mosaic fish. To ascertain whether homozygous mutations could induce sex reversal, we established homozygous mutant lines (Figure S3). By three months post-fertilization, we successfully obtained F2 homozygous mutants for all type I receptor and Smad genes, indicating that none of these mutations result in embryonic lethality. Gonadal histology from three independent batches of XY homozygous mutants for each gene demonstrated that all developed as males with typical testes (Fig. 6). Fluorescence immunohistochemistry further confirmed that their testes expressed Cyp11c1 and Crebt, but not Cyp19a1a or 42Sp50, indicating normal male molecular patterning. These results illustrate that, unlike Alk3 and Smad5, these type I receptors and Smad family members are not essential for male sex determination in Nile tilapia.

### Disruption of *alk3a* or s*mad5* leads to male-to-female sex reversal in Southern catfish

**Figure 7:**
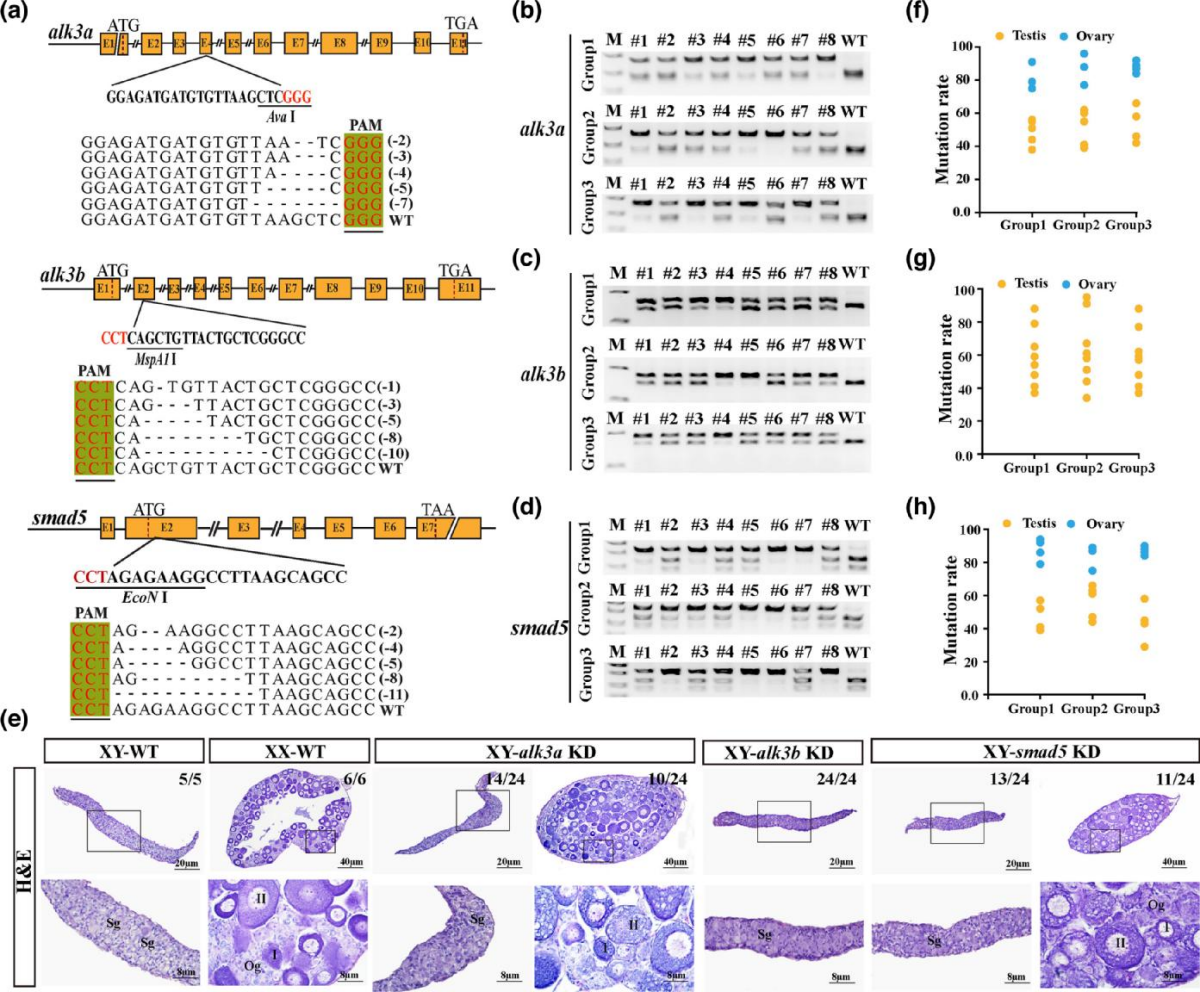
CRISPR/Cas9-mediated knockdown of *alk3a*, *alk3b*, or *smad5* in Southern catfish. a) Schematics of the guide RNA (gRNA) target sites designed for *alk3a*, *alk3b*, and *smad5*, and the mutation types identified in F0 mosaic mutants. (b–d) Genotypic identification of high-efficiency F0 XY mutants. Positive individuals for *alk3a* b), *alk3b* c), and *smad5* d) were identified by restriction enzyme digestion and agarose gel electrophoresis. Mutation screening for each gene was performed in more than three independent biological replicates. e) Histological analysis of gonads from *alk3a*, *alk3b*, and *smad5* mutant XY fish at 90 dpf. Sg, spermatogonia; Og, oogonia; I to II, phase I to II follicle. f–h) Mutation efficiency analysis. Quantification of the mutation rates in the F0 XY mutants for *alk3a* f), *alk3b* g), and *smad5* h).

To assess the functional conservation of Alk3 and Smad5 in the Amhy/Amhr2y sex determination signaling pathway, we generated mutations in *alk3a*, *alk3b*, and *smad5* in Southern catfish, a species in which sex determination is mediated by Amhr2y. Sequencing confirmed various mutations, including both frameshift and nonframeshift types, near the target sites, with at least three independent editing experiments conducted per gene (Fig. 7a). Mutants were screened at 90 dpf using restriction enzymes (*Ava*I for *alk3a*, *MspA1*I for *alk3b*, *EcoN*I for *smad5*; Fig. 7b–d). Gonadal histology was performed on three randomly selected groups, each consisting of eight mutants per gene. A subset of *alk3a* XY mutants (10/24) underwent male-to-female sex reversal, developing ovaries, whereas all *alk3b* XY mutants developed testes. Similarly, a portion of *smad5* XY mutants (11/24) also exhibited sex reversal and formed ovaries (Fig. 7e). Mutation rate analysis revealed gonadal editing efficiencies exceeding 62% in sex-reversed *alk3a* mutants and over 60% in sex-reversed *smad5* mutants (Fig. 7f–h). These results support a conserved role for Alk3 and Smad5 in male sex determination via the Amh/Amhr2 pathway in teleosts.

## Discussion

### Functional specialization of type I receptors and R-Smads in the Bmp signaling pathway

Through systematic screening, this study identified that in Nile tilapia, the combinations of Alk3, Alk6a, or Alk6b with Smad5 significantly enhanced the transduction activity of the Amhy/Amhr2 signal. This finding suggests that despite the increase in the number of type I receptor genes in teleosts due to the third round of whole-genome duplication, their functions in the Amh signaling pathway are not redundant; rather, they exhibit clear functional specialization. Given that the Bmp subfamily encompasses multiple ligands beyond Amh, these type I receptors may also mediate signals from other Bmp ligands. Furthermore, while Alk2 is generally regarded as part of the Bmp signaling pathway, it can also engage in the Activin signaling pathway under specific conditions. Research has shown that Alk2 is the sole member among the 7 TGF-β type I receptors capable of interacting with both Activin and Bmp ligands (Nickel et al. 2019). Notably, even for the same ligand, the type I receptors involved may vary depending on the cell types or biological process (Katagiri et al. 2016). A series of studies has established that Alk3 and Alk6 are significant type I receptors in the Bmp signaling pathway (Zhang et al. 2020). They typically bind to Bmp ligands and play critical roles in various physiological processes, including embryonic development, bone formation, angiogenesis, and cell differentiation. In contrast, Alk2's potential involvement in the Activin signaling pathway could elucidate its limited capacity to effectively activate Smad5 during Amh signal transduction in the present study.

Although no duplicated genes have been identified for the three R-Smad factors (Smad1/5/8) that mediate Bmp signaling in teleost genomes, other R-Smad factors (Smad2/3) that mediate TGF-β subfamily ligand signals possess duplicated genes (Zheng et al. 2018). Similar to type I receptors, Smad1/5/8 serve as intracellular signal transducers for Bmp subfamily ligands, and numerous in vitro studies have demonstrated that different biological processes necessitate the involvement of distinct R-Smads (Zou et al. 2021). In this study, the combinations of Smad5 with Alk3, Alk6a, or Alk6b demonstrated a high responsiveness to Amhy signaling in both HEK293T and SG3 cells. In contrast, activation was observed solely in SG3 cells, specifically when Smad8 was combined with Alk2a. This further supports the notion that the combination of type I receptors and R-Smads in the Amh signaling pathway of teleosts is highly selective. Conversely, in reporter gene assays examining various Smad and type I receptor pairings, we observed that the overexpression of certain receptors led to the suppression of R-Smad phosphorylation. This observation is consistent with findings from murine in vitro studies (Visser 2003), which demonstrate that the increased expression of type I receptors does not universally induce the activation of Smad phosphorylation.

### Functional redundancy and specificity of Alk3 and Smad5 in the Amh/Amhr2 signaling pathway

In Nile tilapia, although there is no sexual dimorphism in the transcription and translation of *alk3* and *smad5* during the critical sex-determination period, the activation of Smad5, as indicated by its phosphorylation (p-Smad5), is significantly stronger and occurs earlier in the nuclei of XY germ cells. This finding demonstrates that the Amhy signal primarily acts through post-translational regulation rather than by modulating gene expression. Furthermore, CRISPR/Cas9-mediated mutations in *alk3* or *smad5* result in male-to-female sex reversal in some XY tilapia with high gonadal mutation rate, further confirming the necessity of both genes in the Amhy signaling pathway. Notably, homozygous mutations of *alk3* or *smad5* led to embryonic lethality at the gastrula stage in tilapia, underscoring the indispensable roles of these two genes in early embryonic development. Consistent with this, experiments involving type I receptor inhibitors revealed successive abnormalities and mortality in Nile tilapia larvae. These findings align closely with studies in mammals and fish (Mishina et al. 1995; Chang et al. 1999; Wei et al. 2014; Tajer et al. 2021), suggesting functional conservation of these genes in vertebrate embryonic development and pluripotency. The lethal phenotype observed in the *alk3* and *smad5* mutants may be attributed to the role of the Bmp signaling pathway in early developmental processes, including mesoderm induction, gastrulation, and cell fate determination (Morgani et al. 2020; Camacho-Aguilar et al. 2024).

This study reveals that the Alk3-Smad5 pair serves as the predominant type I receptor–R-Smad combination in the Amhy/Amhr2y signaling pathway in both Nile tilapia and Southern catfish. This finding aligns with mammalian studies indicating that the Amh signaling pathway inhibits Müllerian duct development, specifically identifying Alk3 as the key type I receptor for this process (Jamin et al. 2002). However, unlike in mammals, where Alk2 and Alk6 may exert compensatory roles across different cell types or physiological contexts (Belville et al. 2005; Beck et al. 2016), mutations in other type I receptors (e.g. Alk2a, Alk2b, Alk6a, Alk6b) in this study did not induce sex reversal. This indicates that Alk3 functions as the specific type I receptor in the Amhy-mediated sex-determination pathway of Nile tilapia. Notably, due to teleost-specific genome duplication events, type I receptor genes exist in multiple copies (Zheng et al. 2018), although the retained copy number varies across species. For instance, the Nile tilapia genome retains a single copy of *alk3*, while the Southern catfish and other teleost genomes contain *alk3a* and *alk3b* (Figure S4). Despite the presence of two Alk3 copies in Southern catfish, mutation of *alk3a* alone is sufficient to induce sex reversal in XY fish, suggesting no functional compensation by the duplicated *alk3b* gene. Beyond its crucial role in sex determination, the Amh/Amhr2 signaling pathway also plays important roles in regulating germ cell proliferation and recruitment (Liu et al. 2020, 2022; di Clemente et al. 2021). Previous research has shown that in zebrafish, the type I receptor mediating Amh signaling for germ cell proliferation is Alk6b (Neumann et al. 2011), further supporting the notion that distinct type I receptors are employed for different biological processes in teleost Amh signal transduction.

In mice, the key R-Smad involved in the Amh signaling pathway for Müllerian duct regression is Smad5; however, Smad1 and Smad8 also exhibit compensatory effects without clear specificity (Orvis et al. 2008). Interestingly, no duplicated copies of Smad1/5/8 genes have been identified in teleost genomes (Figure S5). This study demonstrates that Smad5 serves as the specific R-Smad in the Amhy/Amhr2y sex-determination pathway in both Nile tilapia and Southern catfish. Consequently, the Alk3-Smad5 axis constitutes the exclusive and essential pathway for Amhy/Amhr2y-mediated sex determination in these species, with no functional compensation mechanism. This finding sharply contrasts with the functional complementarity observed among type I receptors or R-Smads in mammals, a difference that may be attributed to the unique nature of sex determination signal transduction mechanisms.

## Conclusions and perspectives

**Figure 8:**
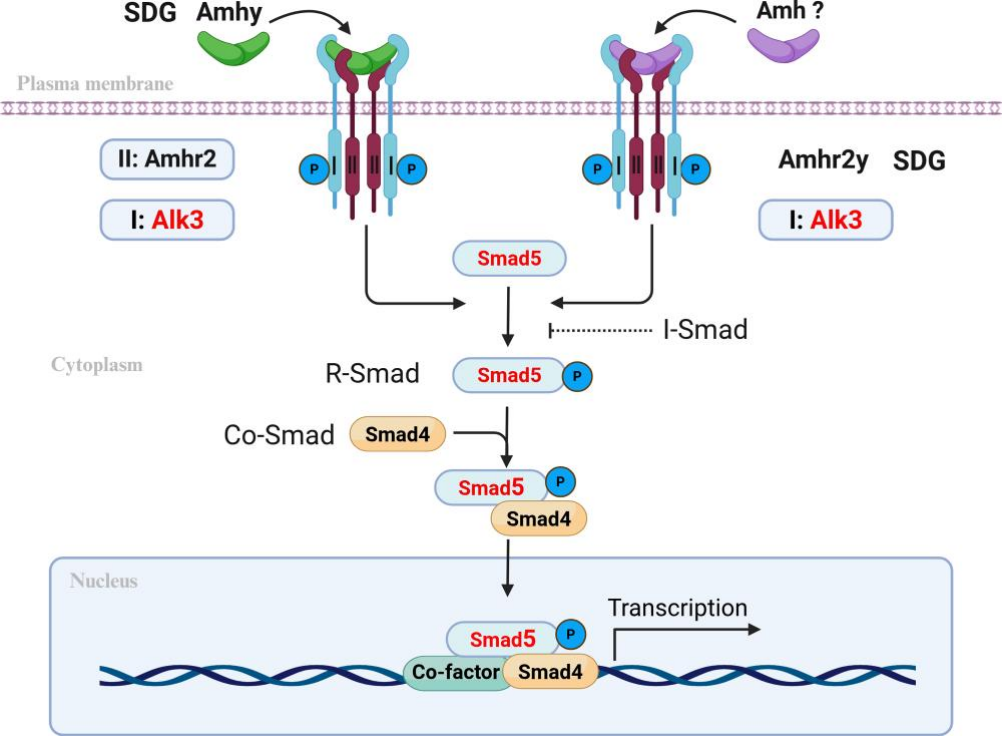
Schematic model of the core Amh signal transduction pathway in teleost sex determination. This model proposes that Alk3 and Smad5 form a conserved core signaling module for male sex determination in teleosts with Amh or Amhr2 homologs as SDG. Despite variation in the initial triggers (SDGs: Amhy or Amhr2y), the signal is transduced through the type I receptor Alk3 (or a specialized paralog in species with gene duplication), leading to the specific phosphorylation and activation of the R-Smad factor Smad5, which is essential for directing testicular development. SDG, Sex-determining gene; II, type II receptor; I, type I receptor; I-Smad, Inhibitory Smad.

In summary, our study has identified a specific sex-determination signal transduction pathway composed of Amh/Amhr2–Alk3–Smad5 in both Nile tilapia and Southern catfish (Fig. 8). We propose that this signaling pathway is likely conserved among teleosts that utilize either Amh or Amhr2 homologs as the SDG. This discovery enhances the understanding of the molecular mechanisms underlying sex determination in teleosts and provides a significant theoretical foundation for further investigations into the evolution and adaptation of this signaling pathway across various species. Future studies that focus on the functions of type I receptors and R-Smad factors in a broader range of teleost species will be essential for fully elucidating the conservation and diversity of the Amh/Amhr2 signaling pathway.

## Materials and methods

### Experimental animals

In this study, Nile tilapia (*Oreochromis niloticus*) and Southern catfish (*Silurus meridionalis*) were maintained in freshwater recirculating aquariums, with the water temperature stabilized at 26 °C and a photoperiod of 12 h light followed by 12 h dark. The fish were fed commercial feed (Shengshuo, China) three times daily. Under these rearing conditions, individuals with XX sex chromosomes developed as females, while those with XY sex chromosomes developed as males.

### DNA extraction, genotyping, and genetic sex identification

Genomic DNA (gDNA) was isolated from caudal fin clips. Briefly, tissue samples were digested with proteinase K, and gDNA was purified using the phenol-chloroform method. The quality and integrity of the gDNA were assessed through agarose gel electrophoresis, and its concentration was measured using a NanoDrop 2000 spectrophotometer (Thermo Fisher Scientific, USA). The gDNA was subsequently diluted to a working concentration of 100 ng/μL for further analyses. Target gene fragments were amplified via PCR. The resulting products were purified using the QIAquick PCR Purification Kit (QIAGEN, Germany) in accordance with the manufacturer's protocol. Genotyping of wild-type, heterozygous, and homozygous mutants was performed either by restriction enzyme digestion or by heteroduplex mobility assay (HMA), followed by confirmation through Sanger sequencing. The genetic sex (XX/XY) of all experimental fish was determined using previously established sex-specific markers for Nile tilapia (Marker-5) and Southern catfish (Marker-8) (Sun et al. 2014; Zheng et al. 2020). All PCR amplifications were conducted using GoTaq PCR Master Mix (Promega, USA) under the following conditions: initial denaturation at 95 °C for 3 min; 36 cycles of 95 °C for 30 s, annealing at 60 °C for 30 s, and extension at 72 °C for 40 s; and a final extension at 72 °C for 10 min. The primer sequences used in this study are listed in Table S1.

### Drug treatment

To identify the type I receptors mediating the Amhy/Amhr2 sex-determination pathway in Nile tilapia, two Bmp type I receptor inhibitors were utilized: DMH2, a specific antagonist of Alk3/Bmpr1a, and LDN193189, a broad-spectrum inhibitor of Bmp type I receptor transcriptional activity. Newly hatched XY fry were immersed in 200 nM DMH2 or 250 nM LDN193189 from 5 to 15 days post-fertilization (dpf), with these concentrations established based on preliminary dose-response tests. From 15 to 30 dpf, the fish were fed a diet supplemented with the corresponding inhibitor. The control group received a diet sprayed with an equivalent volume of a 95% ethanol vehicle. All fish were subsequently reared on standard commercial feed until sampling at 90 dpf for gonadal phenotype analysis. The entire treatment regimen was independently replicated three times.

### Gene mutations in Nile tilapia and southern catfish

Gene mutations in Nile tilapia and Southern catfish were induced using the CRISPR/Cas9 technique, with specific experimental procedures adapted from the laboratory's prior gene-editing study (Li et al. 2014). Initially, mutation target sites for the genes of interest were designed using the online tool ZIFIT (https://www.crisprscan.org/sequence/), targeting sequences of 18 to 20 base pairs in length. The guide RNA (gRNA) was synthesized via in vitro transcription using the mMESSAGE mMACHINE T7 kit (Thermo Fisher Scientific, USA), while the commercially available TrueCut HiFi Cas9 protein (Thermo Fisher Scientific, USA) was utilized directly. In the injection mixture, the final concentrations of gRNA and Cas9 protein were 500 ng/μL and 100 ng/μL, respectively. Microinjection was performed at the one-cell stage of either Nile tilapia or Southern catfish embryos. Mutation efficiency was assessed 72 h post-injection. Individuals with confirmed mutations were reared until 90 dpf for the identification of their genetic sex and gonadal phenotypic sex. To generate homozygous mutants in Nile tilapia, XY individuals harboring mutations from the F0 generation were crossed with XX-WT individuals to produce the F1 generation. Following selection, F1 individuals with frameshift mutations were raised to sexual maturity and then intercrossed, producing homozygous mutants in the F2 generation.

### Gonadal histological analysis

Gonads were collected from fish at designated time points. Prior to dissection, the fish were anesthetized and euthanized by immersion in 0.16 mg/mL tricaine methanesulfonate (MS-222; Sigma-Aldrich, USA). The gonadal tissues were then fixed in Bouin's solution for 24 h at room temperature. Following fixation, the tissues were dehydrated, embedded in paraffin, and sectioned to a thickness of 5 μm using a Leica microtome (Leica Microsystems, Germany). Tissue sections were stained with hematoxylin and eosin (H&E) as previously described (Wang et al. 2010) and imaged with an Olympus BX53F microscope (Olympus, Japan).

### Immunofluorescence

For immunofluorescence (IF) staining, tissue sections were initially permeabilized with phosphate-buffered saline (PBS) containing 1% Triton X-100 for 10 min, followed by a blocking step in PBS with 5% bovine serum albumin (BSA) for 30 min at room temperature. The sections were then incubated overnight at 4 °C with primary antibodies diluted in 5% BSA/PBS. The rabbit polyclonal antibodies generated against Nile tilapia antigens used in this study included Vasa, 42Sp50, Cyp19a1a, Crebt, and Cyp11c1. These antibodies, produced in our laboratory, have been validated for their specificity and working dilution in previous studies. Polyclonal antibodies targeting Nile tilapia Alk3 and Smad5 were commercially generated by Abmart and Abiotech (China), respectively, using antigens detailed in Table S2. Following the primary antibody incubation, the sections were incubated overnight at 4 °C with Alexa Fluor 488- or Alexa Fluor 594-conjugated goat anti-rabbit secondary antibodies (Thermo Fisher Scientific, USA), diluted 1:500 in blocking buffer. Nuclei were counterstained with 4′, 6-diamidino-2-phenylindole (DAPI) using VECTASHIELD mounting medium (Vector Laboratories, USA). Dual-color immunofluorescence histochemistry was performed using the Alexa Fluor 488 Tyramide SuperBoost Kit and the goat anti-rabbit IgG Kit (Thermo Fisher Scientific, USA). For each genotype, at least three biological replicates were examined, and images were acquired using an Olympus FV3000 confocal microscope (Olympus, Japan).

### RNA extraction, reverse transcription PCR and real-time PCR

Total RNA was isolated from fertilized eggs and gonadal tissues of various genotypes using an RNA extraction kit (QIAGEN, USA). For each genotype, 3 to 5 biological replicates were collected, immediately frozen in liquid nitrogen, and stored until processing. RNA concentration was measured using a NanoDrop 2000 spectrophotometer. cDNA was synthesized from DNase I-treated RNA using the PrimeScript RT Reagent Kit with gDNA Eraser (Takara, China), following the manufacturer's protocol. RT-PCR was conducted to confirm the homozygous mutant genotype. Real-time PCR was performed using Fast SYBR Green Master Mix (Takara, Japan) on a 7500 Fast Real-Time PCR system (Applied Biosystems, USA). The housekeeping gene *β-actin* served as the internal control, demonstrating reliable performance in our previous study (Zhang et al. 2017). The expression levels of target genes were normalized to that of *β-actin*. The relative abundance of mRNA transcripts was calculated using the formula *R* = 2^^−ΔΔCt^, as described previously (Livak and Schmittgen 2001). A minimum of three samples for each genotype were analyzed. All primer sequences used in RT-PCR and Real-time PCR are listed in Table S1.

### UAS-Gal4-smad-AD assay

To investigate the differential activation of Smad proteins by Amh type I receptors, medaka spermatogonial stem cell (SG3; Hong et al. 2004) and HEK293T cells were cultured in 6-well plates and co-transfected with four combinations of plasmids. The first plasmid was an expression vector encoding fusion proteins (Smad1-AD-Gal4-DBD, Smad5-AD-Gal4-DBD, Smad8-AD-Gal4-DBD, Constructed in this study), with a concentration of 300 ng per well. These fusion proteins translocate to the nucleus upon phosphorylation by type I receptors, subsequently activating the UAS-4 promoter through their Gal4 DNA-binding domain. The second plasmid was a reporter vector encoding firefly luciferase (pGL4.31[luc2P/GAL4UAS/Hygro] vector, Promega, USA) regulated by a minimal promoter containing UAS sequences, also at 300 ng per well. The third group comprised expression plasmids for Amh signaling-related genes, including pcDNA3.1-Amh, pcDNA3.1-Amhr2, pcDNA3.1-Alk2a, pcDNA3.1-Alk2b, pcDNA3.1-Alk3, pcDNA3.1-Alk6a, pcDNA3.1-Alk6b (Constructed in this study), and control plasmids, totaling 400 ng per well. The final plasmid was a Renilla luciferase expression vector (Promega, USA) for normalization, with a concentration of 5 ng per well. Twenty-four hours post-transfection, cells were washed twice with PBS and lysed with 75 μL of lysis buffer (from the Dual-Luciferase Reporter Assay Kit, Promega, USA). Luciferase activity was then measured. The firefly luciferase activity of the UAS-luc reporter was quantified using the Dual-Luciferase Reporter Assay System (Promega, USA) and normalized against the activity of the co-transfected Renilla luciferase expression plasmid. The experimental data represent results from at least four independent cell transfections and luciferase activity measurements. Statistical significance was assessed using the Mann–Whitney *U* test (*n* = 5).

### Western blot

Western blot analysis was conducted as previously described (Zhang et al. 2017). Total protein was extracted from XX and XY tilapia gonads collected during the critical period of sex determination. Protein lysates were separated using SDS-PAGE on 12% Tris-glycine gels and subsequently transferred onto PVDF membranes. The membranes were blocked for 1 h at room temperature with TBST (10 mM Tris, pH 7.9, 150 mM NaCl, 0.1% Tween-20) containing 5% BSA. Following this, the membranes were incubated overnight at 4 °C with primary antibodies against Alk3, Smad5, and p-Smad5, all diluted to 1:300. After washing with TBST, the membranes were incubated for 1 h at room temperature with HRP-conjugated secondary antibodies (Thermo Fisher Scientific, USA) diluted 1:1,000 in blocking buffer. α-Tubulin, detected using a rabbit anti-α-Tubulin antibody (Cell Signaling Technology, USA) at a dilution of 1:1,000, served as the loading control. Protein signals were developed using Pierce ECL Western Blotting Substrate (Thermo Fisher Scientific, USA) and imaged with a Fusion FX7 system (Vilber Lourmat, France).

### Statistical analysis

All experiments were conducted a minimum of three times to ensure reliability. Data were analyzed using GraphPad Prism 8 software and are presented as mean ± standard deviation (mean ± SD). Differences among multiple groups were assessed using one-way ANOVA, followed by Tukey's multiple comparison test to evaluate intergroup differences. In all statistical analyses, a *P*-value of < 0.05 was deemed statistically significant, with differences indicated by distinct letters in the figures.

## Supplementary Material

msag038_Supplementary_Data

## Data Availability

All data are available in the main text or the Materials.
