# Supplementary material for: Amhy/Amhr2y-mediated sex determination in two distantly related teleosts relies on the conserved Alk3-Smad5 axis
Source: Mol Biol Evol. 2026 Feb 9;43(2):msag038. doi: 10.1093/molbev/msag038 (PMC12925972; doi:10.1093/molbev/msag038)
Supplement: msag038_Supplementary_Data [file msag038_supplementary_data.pdf]

Fig. S1 Expression profiles of additional *alk* and *R-smad* genes

Transcriptomic analysis (Tao et al., 2013) combined with real-time PCR validation revealed the expression patterns of additional *alk* and *R-smad* genes in the gonads during the critical sex determination period (8-15 dpf). No sexually dimorphic mRNA expression was detected between XX (female) and XY (male) fish at these time points.

Fig. S2 The expression of p-Smad5 after *alk3* knockdown.

Western blot analysis and quantitative statistics of phosphorylated Smad5 (p-Smad5) protein expression were conducted on gonadal samples from 1-month-old XY-WT and XY-*alk3* KD groups. (A) The Western blot results demonstrate the expression levels of p-Smad5 in the gonads of both XY-WT and XY-*alk3* KD groups, using α-Tubulin as the loading control. (B) The quantitative analysis reveals the relative percentage of p-Smad5 protein levels normalized to α-Tubulin. The ** symbol indicates a highly significant statistical difference between the two groups ($p < 0.01$).

Fig. S3 Generation and validation of homozygous mutants for additional Amh pathway components.

(A-F) Schematic overview of the CRISPR/Cas9-targeted mutagenesis for type I receptors (*alk2a*, *alk2b*, *alk6a*, *alk6b*) and R-Smad factors (*smad1*, *smad8*). For each gene, the target site and the spectrum of induced mutations are shown. Homozygous mutant lines were successfully established in the F2 generation. The genotypes were confirmed using a combination of Sanger sequencing, polyacrylamide gel electrophoresis (PAGE), restriction enzyme digestion assays, and RT-PCR analysis.

Fig. S4 Phylogenetic analysis of Bmp type I receptors across species.

The phylogenetic tree was reconstructed using the Maximum Likelihood (ML) method in MEGA 6.0 software. Amino acid sequences of the TGF-β peptide domains were aligned with BioEdit. The analysis

reveals that teleost fishes possess varying numbers of receptor copies due to gene duplication. Among the three receptor types, Alk3 and Alk6 form a distinct clade, indicating a close evolutionary relationship and shared ancestry. Sequence accession numbers are listed in Table S2.

Fig. S5 Phylogenetic analysis of Bmp R-Smad proteins across species.

The phylogenetic tree was constructed using the same Maximum Likelihood method as described for Fig. S2. Although teleost fishes have undergone an additional round of whole-genome duplication (3R), each R-Smad gene (*smad1*, *smad5*, *smad8*) is typically maintained as a single copy, suggesting potential subfunctionalization. The tree topology reveals that Smad1 and Smad5 form a distinct clade, indicating a closer evolutionary relationship between them compared to Smad8. GenBank accession numbers are listed in Table S2.

Fig. S1

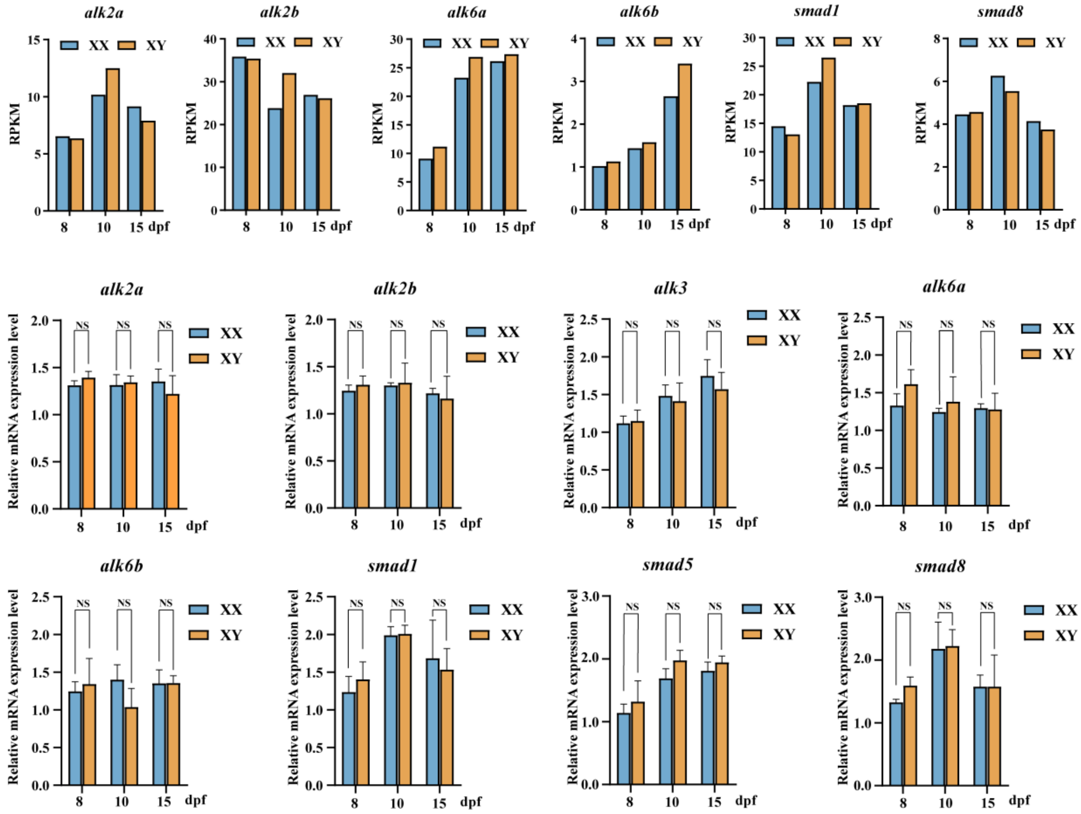

alk2a
alk2b
alk6a
alk6b
smad1
smad8
XX
XY
RPKM
8
10
15 dpf
alk2a
alk2b
alk3
alk6a
alk6b
smad1
smad5
smad8
Relative mRNA expression level
NS
dpf

Fig. S2

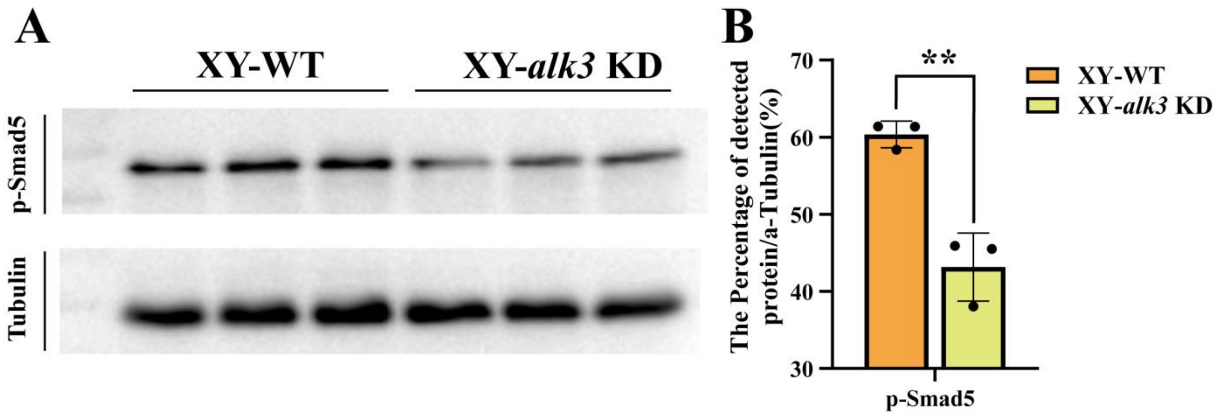
A
XY-WT
XY-*alk3* KD
p-Smad5
Tubulin
B
The Percentage of detected protein/a-Tubulin(%)
70
60
50
40
30
**
p-Smad5
XY-WT
XY-*alk3* KD

Fig. S3

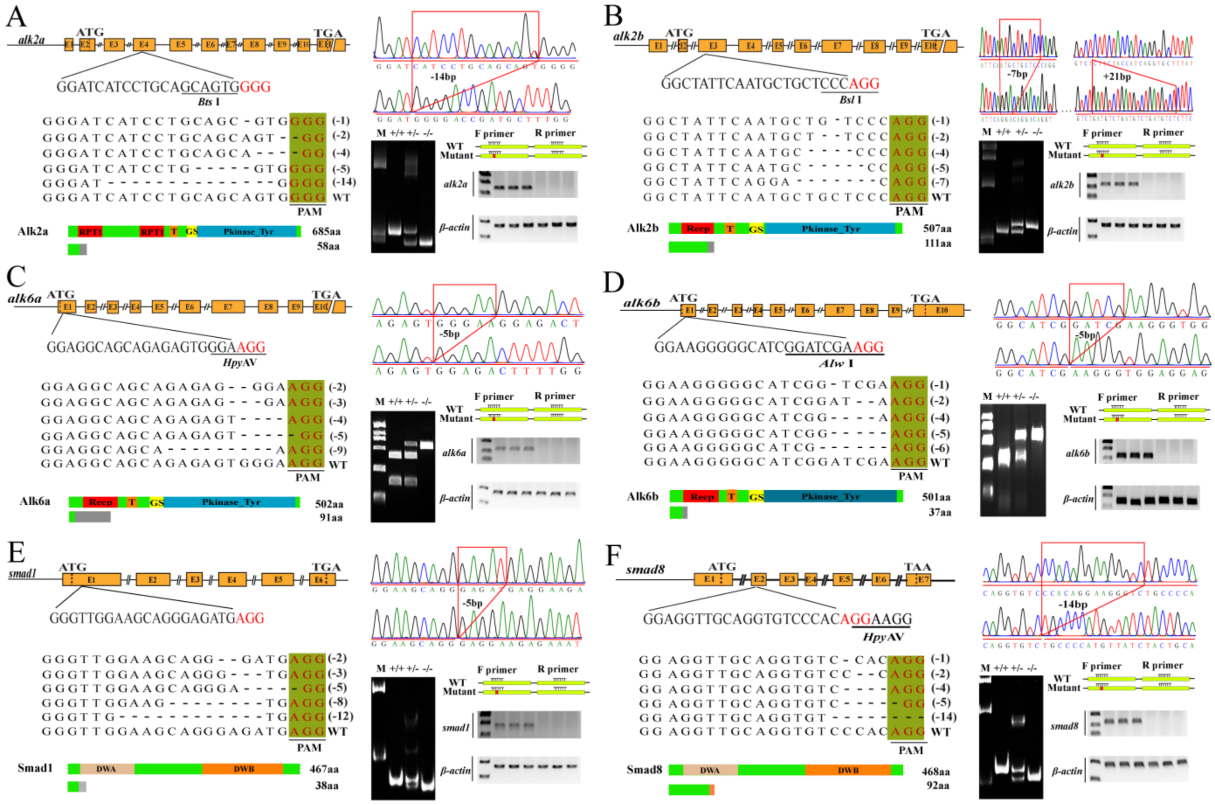

A
alk2a
ATG
TGA
GGATCATCCTGCAGCAGTGGGG
Bts I
-14bp
PAM
Alk2a
685aa
58aa
alk2a
β-actin
F primer
R primer
WT
Mutant
B
alk2b
GGCTATTCAATGCTGCTCCCAGG
Bsl I
-7bp
+21bp
Alk2b
507aa
111aa
C
alk6a
GGAGGCAGCAGAGAGTGGGAAGG
HpyAV
-5bp
Alk6a
502aa
91aa
D
alk6b
GGAAGGGGGCATCGGATCGAAGG
Alw I
Alk6b
501aa
37aa
E
smad1
GGGTTGGAAGCAGGGAGATGAGG
Smad1
467aa
38aa
F
smad8
TAA
GGAGGTTGCAGGTGTCCCACAGGAAGG
HpyAV
Smad8
468aa
92aa

Fig. S4

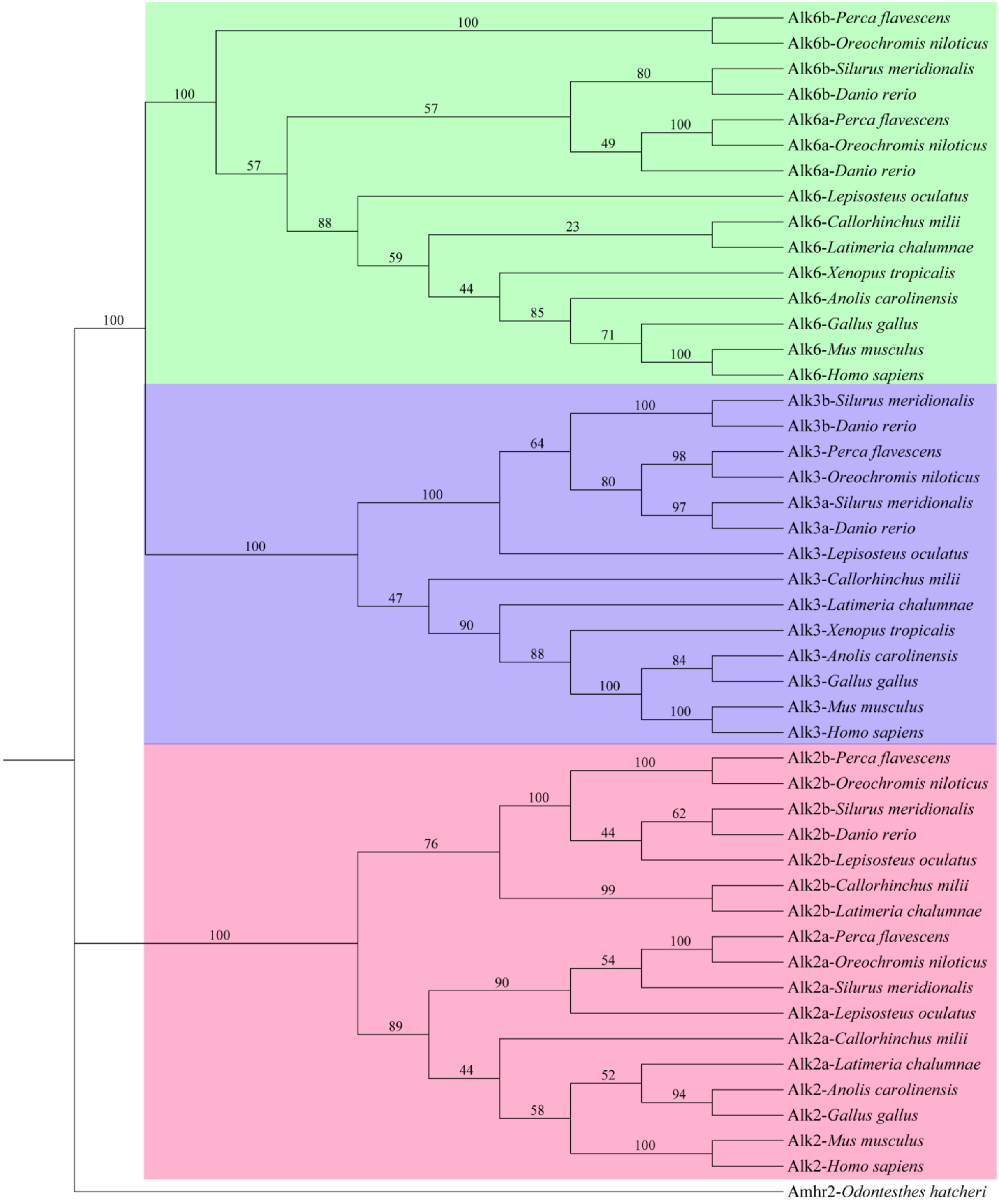
Alk6b-*Perca flavescens*
Alk6b-*Oreochromis niloticus*
Alk6b-*Silurus meridionalis*
Alk6b-*Danio rerio*
Alk6a-*Perca flavescens*
Alk6a-*Oreochromis niloticus*
Alk6a-*Danio rerio*
Alk6-*Lepisosteus oculatus*
Alk6-*Callorhinchus milii*
Alk6-*Latimeria chalumnae*
Alk6-*Xenopus tropicalis*
Alk6-*Anolis carolinensis*
Alk6-*Gallus gallus*
Alk6-*Mus musculus*
Alk6-*Homo sapiens*
Alk3b-*Silurus meridionalis*
Alk3b-*Danio rerio*
Alk3-*Perca flavescens*
Alk3-*Oreochromis niloticus*
Alk3a-*Silurus meridionalis*
Alk3a-*Danio rerio*
Alk3-*Lepisosteus oculatus*
Alk3-*Callorhinchus milii*
Alk3-*Latimeria chalumnae*
Alk3-*Xenopus tropicalis*
Alk3-*Anolis carolinensis*
Alk3-*Gallus gallus*
Alk3-*Mus musculus*
Alk3-*Homo sapiens*
Alk2b-*Perca flavescens*
Alk2b-*Oreochromis niloticus*
Alk2b-*Silurus meridionalis*
Alk2b-*Danio rerio*
Alk2b-*Lepisosteus oculatus*
Alk2b-*Callorhinchus milii*
Alk2b-*Latimeria chalumnae*
Alk2a-*Perca flavescens*
Alk2a-*Oreochromis niloticus*
Alk2a-*Silurus meridionalis*
Alk2a-*Lepisosteus oculatus*
Alk2a-*Callorhinchus milii*
Alk2a-*Latimeria chalumnae*
Alk2-*Anolis carolinensis*
Alk2-*Gallus gallus*
Alk2-*Mus musculus*
Alk2-*Homo sapiens*
Amhr2-*Odontesthes hatcheri*

Fig. S5

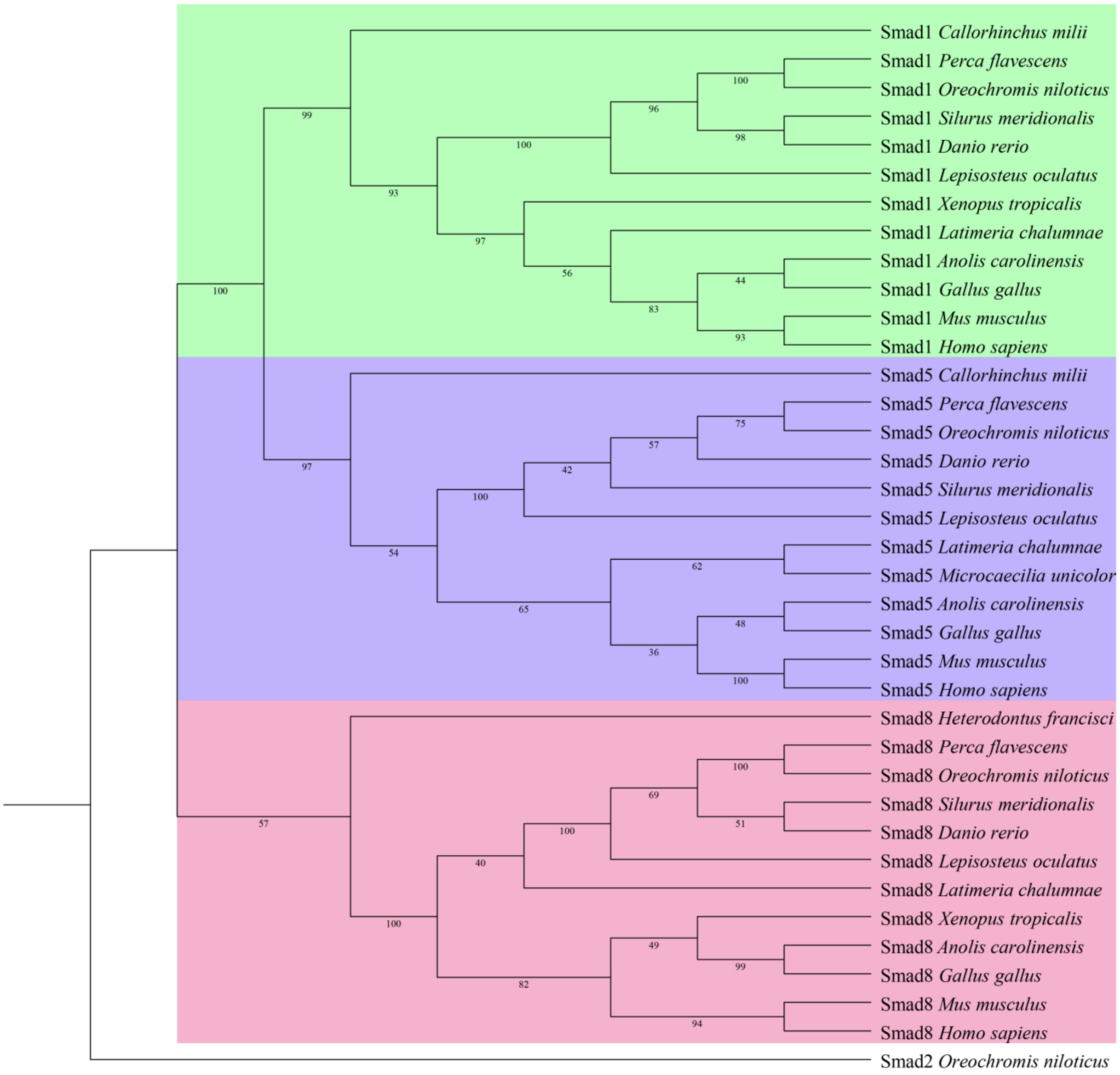
Smad1 *Callorhinchus milii*
Smad1 *Perca flavescens*
Smad1 *Oreochromis niloticus*
Smad1 *Silurus meridionalis*
Smad1 *Danio rerio*
Smad1 *Lepisosteus oculatus*
Smad1 *Xenopus tropicalis*
Smad1 *Latimeria chalumnae*
Smad1 *Anolis carolinensis*
Smad1 *Gallus gallus*
Smad1 *Mus musculus*
Smad1 *Homo sapiens*
Smad5 *Callorhinchus milii*
Smad5 *Perca flavescens*
Smad5 *Oreochromis niloticus*
Smad5 *Danio rerio*
Smad5 *Silurus meridionalis*
Smad5 *Lepisosteus oculatus*
Smad5 *Latimeria chalumnae*
Smad5 *Microcaecilia unicolor*
Smad5 *Anolis carolinensis*
Smad5 *Gallus gallus*
Smad5 *Mus musculus*
Smad5 *Homo sapiens*
Smad8 *Heterodontus francisci*
Smad8 *Perca flavescens*
Smad8 *Oreochromis niloticus*
Smad8 *Silurus meridionalis*
Smad8 *Danio rerio*
Smad8 *Lepisosteus oculatus*
Smad8 *Latimeria chalumnae*
Smad8 *Xenopus tropicalis*
Smad8 *Anolis carolinensis*
Smad8 *Gallus gallus*
Smad8 *Mus musculus*
Smad8 *Homo sapiens*
Smad2 *Oreochromis niloticus*

Table S1. Primers used in this study

Primer name	Sequence (5'-3')	Purpose
ON-alk2a-gRNA-F	TAATACGACTCACTATAGGATCATCCTGCAGCAGTGGTTTTAGAGCTAGAAATAGC	CRISPR/Cas9
ON-alk2b-gRNA-F	TAATACGACTCACTATAGGCTATTCAATGCTGCTCCCGTTTTAGAGCTAGAAATAGC	
ON-alk3-gRNA-F	TAATACGACTCACTATAGGGGACGGGGGTGAAACCCGGTTTTAGAGCTAGAAATAGC	
ON-alk6a-gRNA-F	TAATACGACTCACTATAGGAGGCAGCAGAGAGTGGGAGTTTTAGAGCTAGAAATAGC	
ON-alk6b-gRNA-F	TAATACGACTCACTATAGGAAGGGGGCATCGGATCGAGTTTTAGAGCTAGAAATAGC	
ON-smad1-gRNA-F	TAATACGACTCACTATAGGGTTGGAAGCAGGGAGATGGTTTTAGAGCTAGAAATAGC	
ON-smad5-gRNA-F	TAATACGACTCACTATAGGATACTCGCAAATGTCCAGGTTTTAGAGCTAGAAATAGC	
ON-smad8-gRNA-F	TAATACGACTCACTATAGGAGGTTGCAGGTGTCCCACGTTTTAGAGCTAGAAATAGC	
SM-alk3a-gRNA-F	TAATACGACTCACTATAGGAGATGATGTGTTAAGCTCGTTTTAGAGCTAGAAATAGC	
SM-alk3b-gRNA-F	TAATACGACTCACTATAGGCCCGAGCAGTAACAGCTGGTTTTAGAGCTAGAAATAGC	
SM-smad5-gRNA-F	TAATACGACTCACTATAGGCTGCTTAAGGCCTTCTCTGTTTTAGAGCTAGAAATAGC	
gRNA-R	AGCACCGACTCGGTGCCAC	

ON-alk2a-page-F	TGTTTCCTGTTGCAGGTGAG	Mutant screening
ON-alk2a-page-R	AGCTGAAGCACTCCTGACCA	
ON-alk2b-page-F	CCTTCTCCAGTGCTTCACAG	
ON-alk2b-page-R	CTGATGGTAGAAGAGACATC	
ON-alk3-page-F	CGTGTCCAGCTGCAGGTCAG	
ON-alk3-page-R	CGATGGTGGGACCGTCGCCA	
ON-alk6a-page-F	CTCTGTTGTATAGGCAAC	
ON-alk6a-page-R	CACAGGAGTCTTTGGGAGG	
ON-alk6b-page-F	CCTGTGTCTTCACTGTACAGC	
ON-alk6b-page-R	AGTGGTGGTAGCAGTGGCACC	
ON-smad1-page-F	GTGAAACGGCTGCTGGGTTG	
ON-smad1-page-R	ACCAGAGCATCCACTGCCT	
ON-smad5-page-F	GCTGGCCGGATCTACAGTCC	
ON-smad5-page-R	GCACACCTCCTTCTGTTTGG	
ON-smad8-page-F	CCTCCAGGTAAGTGTGTGAC	
ON-smad8-page-R	GCTCATGGTGGGACTGCAAG	
ON-alk2a-test-F	CAAGTAGCAGGCATTGATCA	
ON-alk2a-test-R	GGACTAAGGAGTCAGCTACC	
ON-alk2b-test-F	GTGGGTTTAAAGTATTGTG	
ON-alk2b-test-R	CACCTTCTGGGGCTGTGGGT	
ON-alk3-test-F	CTGCTCAGCGATCTCTGACC	
ON-alk3-test-R	CAGCCGGAGGTGAGCTGCAC	
ON-alk6a-test-F	GCAAGCCTACTCTCTAGCAATG	
ON-alk6a-test-R	TGGAAGGATTTGTGCCAGATGA	

ON-alk6b-test-F	GAGGCATGTTGCTGCCAACA	
ON-alk6b-test-R	GCTGTGCCTCACGAAGCTTC	
ON-smad1-test-F	CTTGCATCTTGGAAGCCGAGGAT	
ON-smad1-test-R	GGGATTTCACTCACCTGGACTGTC	
ON-smad5-test-F	CCATTCCAAGATCACTGGACGG	
ON-smad5-test-R	GTGATGACTATGCCTCACAG	
ON-smad8-test-F	GCAACATACACACATGCGTGC	
ON-smad8-test-R	CAGATGTCCTTCTGCTTGGAG	
SM-alk3a-page-F	GGTCAGTGCTTTGCCATCATTG	
SM-alk3a-page-R	CACTGAAAATGGGAGCCTTC	
SM-alk3b-page-F	TCCATGGCACGGGAATGAAAC	
SM-alk3b-page-R	GTTGTTGGTGGCATCCTCTGG	
SM-smad5-page-F	GGATGCGCTGGTGAAGAAGC	
SM-smad5-page-R	ATGGTCACACACTTGCTGG	
SM-alk3a-test-F	GTCACTGCCCAGAAGATGCC	
SM-alk3a-test-R	GCTTCAGATCACGGTTGCAG	
SM-alk3b-test-F	TCCATGGCACGGGAATGAAAC	
SM-alk3b-test-R	ACCTTGCACTGGAAGTGGGA	
SM-smad5-test-F	GTCAAACCCGACTAGCAGTC	
SM-smad5-test-R	TCTGGAACACTGCTTCATAGC	
ON-alk2a-RT-F	CGGATCATCCTGCAGCAGTG	RT-PCR
ON-alk2a-RT-R	AGTGCTGGCCTGTGCAGCGA	
ON-alk2b-RT-F	GGCTATTCAATGCTGCTCCC	
ON-alk2b-RT-R	CCGTCTATGGCTCCCTGCTC	

ON-alk3-RT-F	GGGTGAAACCCGAGGCCCGG	
ON-alk3-RT-R	GCTGCAGGTCTCTGTTGCAC	
ON-alk6a-RT-F	GCAGAGAGTGGGAAGGAGAC	
ON-alk6a-RT-R	TCGCCTTTGACGTGAGTTCC	
ON-alk6b-RT-F	CATCGGATCGAAGGGTGGAG	
ON-alk6b-RT-R	AGAGCTCTCCTTGAACGTGC	
ON-smad1-RT-F	GCAGGGAGATGAGGAAGAGA	
ON-smad1-RT-R	ACTCGCTGTTCCTCGGTACC	
ON-smad5-RT-F	AGCCTCTGGACATTTGCGAG	
ON-smad5-RT-R	CTGGCCGATTGGCTCATCAG	
ON-smad8-RT-F	GGTGTCCCACAGGAAGGGTC	
ON-smad8-RT-R	TGTGTTGAGGGTTGAACTCG	
ON-alk2a-qPCR-F	TACTGCTGCCAGGGACACCT	Real-time PCR
ON-alk2a-qPCR-R	CACCAGGATAGCGACAGCAG	
ON-alk2b-qPCR-F	TGAGCAGGGAGCCATAGACG	
ON-alk2b-qPCR-R	CCTACGCACTCCACCAGACT	
ON-alk3-qPCR-F	AGGACCAGGAGCATGATGTC	
ON-alk3-qPCR-R	CGATCTGACGCACCATCTGG	
ON-alk6a-qPCR-F	AAGCTCAGGCTCAGGCTCAG	
ON-alk6a-qPCR-R	CGCTTCCTCAGTGGTGAAGA	
ON-alk6b-qPCR-F	GCAGCATTGGGTCTGGCTCT	
ON-alk6b-qPCR-R	CTCTCTCCTCTCCACTTGCC	
ON-smad1-qPCR-F	CCCACCATGAGCTGAAAGCG	
ON-smad1-qPCR-R	ACTCGCTGTTCCTCGGTACC	

ON-smad5-qPCR-F	CTGATGAGCCAATCGGCCAG	
ON-smad5-qPCR-R	TGGTAAGCCTCTCCGACACG	
ON-smad8-qPCR-F	CCTTCGGCTCCAAGCAGAAGG	
ON-smad8-qPCR-R	AGGCTCGTTGTGAAGGGAGG	
ON-marker-F5	ATGGCTCCGAGACCTTGACTG	Genetic sex identification
ON-marker-R3	CAGAAATGTAGACGCCCAGGTAT	
SM-marker-F8	ATGGCTCCGAGACCTTGACTG	
SM-marker-R8	TGTGTTCATCAGCCATAAGGGTGTT	
ON-alk2a-pcDNA3.1-F	GCGTTTAAACTTAAGCTTGGTACCGAGCTCAT GACGAGTGTGGACATGTTTC	Luciferase
ON-alk2a-pcDNA3.1-R	CGAGGCTGATCAGCGGGTTTAAACGGGCCCT CAAATGTCTGTTTTTATTTTATCC	
ON-alk2b-pcDNA3.1-F	GCGTTTAAACTTAAGCTTGGTACCGAGCTCAT GGGTCGTTGCAGTGTCCA	
ON-alk2b-pcDNA3.1-R	CGAGGCTGATCAGCGGGTTTAAACGGGCCCT CACGACTCCTTGCCCTTTTCC	
ON-alk3-pcDNA3.1-F	GCGTTTAAACTTAAGCTTGGTACCGAGCTCAT GCCAGGTCTACCGGTCTG	
ON-alk3-pcDNA3.1-R	CGAGGCTGATCAGCGGGTTTAAACGGGCCCT CAGATCTTGATGTCCTGGG	
ON-alk6a-pcDNA3.1-F	GCGTTTAAACTTAAGCTTGGTACCGAGCTCAT GCTGCTAAAAGCATCCAG	
ON-alk6a-pcDNA3.1-R	CGAGGCTGATCAGCGGGTTTAAACGGGCCCT CACAGCTTGATATCCTGTG	

ON-alk6b-pcDNA3.1-F	GCGTTTAAACTTAAGCTTGGTACCGAGCTCATGCTCCTGAAGAACGGGGG	
ON-alk6b-pcDNA3.1-R	CGAGGCTGATCAGCGGGTTTAAACGGGCCCTCACAGTTTGATATCTTGAG	
gal4-3.1-F	GCGTTTAAACTTAAGCTTGGTACCGAGCTCATGAAGCTACTGTCTTCTATC	
gal4-smad1-3.1-F	GGTCAAAGACAGTTGACTGTATCGATGAATGTCACTTCGCTCTTC	
gal4-smad1-3.1-R	GAAGAGCGAAGTGACATTCATCGATACAGTCAACTGTCTTTGACC	
gal4-smad5-3.1-F	GGTCAAAGACAGTTGACTGTATCGATGACTTCCATGTCTAGTTTG	
gal4-smad5-3.1-R	CAAACTAGACATGGAAGTCATCGATACAGTCAACTGTCTTTGACC	
gal4-smad8-3.1-F	GGTCAAAGACAGTTGACTGTATCGATGATTGAAACTTGACCTGG	
gal4-smad8-3.1-R	CCAGGTCAAGTTTCAATCATCGATACAGTCAACTGTCTTTGACC	
smad1-3.1-R	CGAGGCTGATCAGCGGGTTTAAACGGGCCCCTAGGAGACGGAGGATATGG	
Smad5-3.1-R	CGAGGCTGATCAGCGGGTTTAAACGGGCCCTTAGGACACAGAGGAGATTG	
Smad8-3.1-R	CGAGGCTGATCAGCGGGTTTAAACGGGCCCTTACGACACTGAAGAAATTGGG	

F, forward primer; R, reverse primer; ON, *Oreochromis niloticus*; SM, *Silurus meridionalis*

Table S2. Detailed information on antigens

Alk3	Ac-NPDHVLOGTGVKPEARRPGDGPTIAPEDLPRSLSC-NH2
Smad5	Ac-<br>EEPSHWCSIVYYELNNRVGEAYHASSTSVLVDGFTDPSNN<br>KNRFCLGLLSNVNRNSTIENTRRHIGKGVHLYYVGGEVYA<br>ECLSDTSIFVQSRNCNYHHGFHPTTVCKIPSGCSLKIFNNQE<br>FAQLLAQSVNHGFEAVYELTKMCTIRMSFVKGWGAEYH<br>RQDVTSTPCWIEVHLHGPLQWLDKVLTQMGSPLNPISSVS-<br>NH2
